# *LncRNA-TBP* mediates TATA-binding protein recruitment to regulate myogenesis and induce slow-twitch myofibers

**DOI:** 10.1186/s12964-022-01001-3

**Published:** 2023-01-12

**Authors:** Manting Ma, Bolin Cai, Zhen Zhou, Shaofen Kong, Jing Zhang, Haiping Xu, Xiquan Zhang, Qinghua Nie

**Affiliations:** 1 Lingnan Guangdong Laboratory of Modern Agriculture & State Key Laboratory for Conservation and Utilization of Subtropical Agro-Bioresources, College of Animal Science, South China Agricultural University, Guangzhou, 510642 Guangdong China; 2 Guangdong Provincial Key Lab of Agro-Animal Genomics and Molecular Breeding, and Key Laboratory of Chicken Genetics, Breeding and Reproduction, Ministry of Agriculture, Guangzhou, 510642 Guangdong China

**Keywords:** *LncRNA-TBP*, RNA binding proteins (RBPs), TBP, Myogenesis, Muscle phenotype transformation

## Abstract

**Background:**

Skeletal muscle is comprised of heterogeneous myofibers that differ in their physiological and metabolic parameters. Of these, slow-twitch (type I; oxidative) myofibers have more myoglobin, more mitochondria, and higher activity of oxidative metabolic enzymes compared to fast-twitch (type II; glycolytic) myofibers.

**Methods:**

In our previous study, we found a novel *LncRNA-TBP* (for “LncRNA directly binds TBP transcription factor”) is specifically enriched in the soleus (which has a higher proportion of slow myofibers). The primary myoblast cells and animal model were used to assess the biological function of the *LncRNA-TBP *in vitro or in vivo. Meanwhile, we performed a RNA immunoprecipitation (RIP) and pull-down analysis to validate this interaction between *LncRNA-TBP* and TBP.

**Results:**

Functional studies demonstrated that *LncRNA-TBP* inhibits myoblast proliferation but promotes myogenic differentiation in vitro. In vivo, *LncRNA-TBP* reduces fat deposition, activating slow-twitch muscle phenotype and inducing muscle hypertrophy. Mechanistically, *LncRNA-TBP* acts as a regulatory RNA that directly interacts with TBP protein to regulate the transcriptional activity of TBP-target genes (such as *KLF4*, *GPI*, *TNNI2*, and *CDKN1A*).

**Conclusion:**

Our findings present a novel model about the regulation of *LncRNA-TBP*, which can regulate the transcriptional activity of TBP-target genes by recruiting TBP protein, thus modulating myogenesis progression and inducing slow-twitch fibers.

**Video Abstract**

**Supplementary Information:**

The online version contains supplementary material available at 10.1186/s12964-022-01001-3.

## Background

Skeletal muscles constitute approximately 35% of the body weight and main resources of animal protein for human consumption. Abnormal regulation of skeletal muscle‐specific genes leads to various muscle diseases that development directly influences animal meat quantity and quality [1–3]. Myogenesis is a highly ordered process during which muscle stem cell proliferation, migration, differentiation, and fusion are activated to form myofibers [4].

Skeletal muscle is composed of different types of myofibers, which are mainly differentiated by the expression of heavy chain myosin and the reliance on oxidative phosphorylation [5]. Compared to fast-twitch (type II; glycolytic) myofibers, slow-twitch (type I; oxidative) myofibers have more myoglobin, more mitochondria, and higher activity of oxidative metabolic enzymes [6, 7]. Under certain conditions, fast-twitch myofibers and slow-twitch myofibers can transform into each other [8].

Transcriptional activation is a major step in the regulation of gene expression during development. TATA-binding protein (TBP) and 12–15 associated factors (TAFs) together form the pre-initiation complex (PIC), which as an obligate step leading to transcription [9, 10]. In mice, a large polyQ repeat in TBP causes primary muscle degeneration and decreases the association of MyoD with TBP and DNA promoters [11]. Recent studies have demonstrated that the TBP associated factor (TAF9b) plays an important role in neurodevelopmental and mental disorders. As a coactivator that stabilized the structure of P53, it also participates in P53-mediated apoptosis and cell cycle regulation [12–15].

Long noncoding RNAs (lncRNAs), a novel class of regulatory RNAs, are commonly defined as transcribed RNAs with sizes ranging from 200 bp to > 100 kb and not translated to protein [16]. Substantial evidences have shown that lncRNAs play critical regulatory roles in diverse biological processes and diseases, such as skeletal muscle development and muscle disorders [17, 18]. Based on our previous RNA-sequencing (RNA-seq) analysis, we screened and identified an lncRNA (MSTRG.6038.1), which differentially expressed between pectoralis major (PEM, which is mainly composed of fast-twitch fibers) and soleus (SOL, which has a higher proportion of slow muscle fibers) in 7-week-old Xinghua chicken, was named “*LncRNA-TBP*” (for “LncRNA directly binds TBP transcription factor”). Functional studies demonstrated that *LncRNA-TBP* inhibits myoblast proliferation but promotes myogenic differentiation as well as reduces fat deposition, activating slow-twitch muscle phenotype and reducing muscle atrophy. Mechanistically, *LncRNA-TBP* acts as a regulatory RNA that directly interacts with TBP protein to regulate the transcriptional activity of TBP-target genes (such as *KLF4*, *GPI*, *TNNI2*, and *CDKN1A*).

## Materials and methods

### Ethics statement

The experimental animals were chickens of Chinese local breeds. All animal studies were sanctioned by the Institutional Animal Care and Use Committee at the South China Agricultural University. All the experiments were performed according to the regulations and guidelines established by the committee and international standards for animal welfare (approval ID: SCAU#2021c008). We made every effort to reduce the suffering of animals.

### Experimental animals and tissues

Four 7-week-old Xinghua female chickens were received from the Zhicheng Poultry Breeding Co., Ltd. (Guangdong, China). The tissues (including the cerebrum, cerebellum, hypothalamus, heart, liver, spleen, lung, kidney, muscular stomach, glandular stomach, breast muscle, and leg muscle) were collected, quickly frozen into liquid nitrogen, and then stored at − 80 °C.

### Cell culture and transfection

CPMs were isolated from E11 chicken leg muscles as previously described [19] and cultured in Roswell Park Memorial Institute (RPMI)-1640 medium (Gibco, USA) with 20% fetal bovine serum (Gibco). All transient transfections were performed using Lipofectamine 3000 reagent (Invitrogen, USA) according to the manufacturer’s instructions.

### Lentivirus assay

Chinese native breeds (XH chickens) were used for the in vivo experiment in this study. For the construction of animal models of *LncRNA-TBP* overexpression and knockdown.

1-day-old chicks were randomly divided into two groups (Lv-LncRNA-TBP and Lv-NC; n = 30), respectively. Chicks received two intramuscular doses of lentivirus (10^6^ titers) in two different sites of the gastrocnemius. Thirteen days after the initial injection, chick gastrocnemius samples were collected from the above two groups.

7-days-old chickens were randomly divided into two groups (Chol-ASO-LncRNA-TBP and Chol-ASO-NC; n = 15) respectively. Chicks received two intramuscular doses of modified ASOs (40 nmol) by intramuscular injection on days 14 and 18. The chickens were euthanized at 21 days old, and the gastrocnemius muscles were detached and stored at − 80 °C.

### Rapid-amplification of cDNA ends (RACE)

The full-length of *LncRNA-TBP* was amplified by using a SMARTer RACE cDNA Amplification Kit (Clontech, Japan), following the manufacturer’s instructions. The primer pairs used in RACE are listed in Additional file 10: Table S4.

### RNA isolation, complementary DNA (cDNA) synthesis, and real-time (RT) PCR analysis

Total RNA was extracted from tissues or cells using RNAiso plus reagent (TaKaRa, Japan). cDNA synthesis was obtained by using a PrimeScript RT Reagent Kit with gDNA Eraser (Perfect Real Time) (Takara, Japan). Real-time quantitative PCR (qRT-PCR) reactions were performed on a QuantStudio 5 Real-Time PCR Systems (Thermo Fisher, Waltham, MA, USA) by using an ChamQ Universal SYBR qPCR Master Mix (Vazyme, China). All primers for RT-PCR and real-time qPCR are listed in Additional file 10: Table S4.

### Plasmid construction and RNA oligonucleotides

For Flag fusion protein construction, twelve ORFs of *LncRNA-TBP* were amplified and cloned into pcDNA3.1-3xFlag (SiDanSai, Shanghai, China), and pcDNA3.1-3xFlag-β-actin was used as a positive control.

For overexpression vectors construction, the full-length sequence of *LncRNA-TBP* was amplified and cloned into reconstructive pcDNA3.1 vector (Promega, Madison, WI, USA), which with GFP tag at the C-terminus.

For viral vectors constructed, the full-length sequence of *LncRNA-TBP* was amplified and then cloned into the lentiviral vector (pLVX-mCMV-ZsGreen-IRES-Puro; Addgene, Cambridge, MA, USA).

The antisense oligonucleotide (ASO) with Cy3-modified that was used for the specific knockdown of *LncRNA-TBP* was designed and synthesized by Guangzhou RiboBio (Guangzhou, China). The siRNA against *TBP* (NCBI Reference Sequence: NM_205103.1) was also designed and synthesized.

The primers and oligonucleotide sequences used in this study are shown in Additional file 10: Table S4 and S5.

### Flow cytometry, 5-ethynyl-2’-deoxyuridine (EdU) and cell counting kit-8 (CCK-8) assays

The Cell Cycle Analysis Kit (Thermo Fisher Scientific, USA), C10310 EdU Apollo In Vitro Imaging Kit (RiboBio, China) and TransDetect Cell Counting Kit (TransGen, Beijing, China) were used for flow cytometry, EdU, and CCK-8 assay, as the manufacturer’s protocol.

### Immunofluorescence, immunohistochemistry and hematoxylin and eosin staining

Immunofluorescence was performed using anti-MyHC (B103; DHSB, USA; 2.5 mg/mL), and images were captured using a fluorescence microscope (DMi8; Leica, Germany). The area of cells labeled with anti-MyHC was measured and calculated as previously described [20].

Immunohistochemistry was carried out using an SP-POD Kit (SP0041; Solarbio, China) with primary antibodies including anti-MYH1A (F59, DSHB, 1:100) and anti-MYH7B (S58, DSHB, 1:300). The number of myofibers labeled with anti-MYH1A or anti-MYH7B was calculated.

Hematoxylin and eosin (H&E) staining was performed using muscle tissues embedded in paraffin and cut into 4-mm-thick transverse sections. Subsequently, the sections were stained with H&E.

### Mitochondrial DNA (mtDNA) content and fatty acid oxidation (FAO) rate assay

Total DNA was extracted using the Tissue DNA Kit (D3396, Omega, GA, USA) according to the manufacturer’s instructions. The amount of mitochondrial DNA was determined by quantification of cytochrome c oxidase subunit II (COX2). The nuclear-encoded β-globin gene was used as an internal control. Primers used in this study can be found in Additional file 10: Table S4.

The mitochondria of the myoblast and gastrocnemius were isolated using the Cell/Tissue Mitochondria Isolation Kit (C3601/C3606, Beyotime, China). After measuring the mitochondrial protein concentration, freshly isolated mitochondria were subjected to FAO rate assay with the Colorimetric Fatty Acid Oxidation Rate Assay Kit (HL50679, Haling, Shanghai, China), according to the manufacturer’s protocol.

### Metabolite and enzyme activities assays

Content of TG, FFA, and glycogen as well as enzyme activity of LDH and SDH in skeletal muscle were measured using commercially available kits (BC0625, BC0595, BC0345, BC0685, BC0955, respectively; Solarbio, China), according to the manufacturer’s instructions.

### Central carbon metabolic profiling

*LncRNA-TBP* overexpression gastrocnemius samples (n = 6) were used for metabolites extraction and then performed on HPIC-MS/MS analysis. The high-performance ion-exchange liquid chromatography (HPIC) separation was carried out using a Thermo Scientific Dionex ICS-6000 HPIC System (Thermo Fisher Scientific, IL, USA). An AB SCIEX 6500 QTRAP+ triple quadrupole mass spectrometer (AB Sciex, USA), equipped with electrospray ionization (ESI) interface, was applied for assay development.

Metabolic hierarchical clustering analysis (HCA) was performed using Cluster3.0 software as previously described [21].

### Western blot analysis

Western blot analysis was performed as previously described [44]. The primary antibodies used were anti-FLAG (AF519, Beyotime,1:1,000), anti-MYOD (ABP53067, Abbkine, 1:500), anti-MyHC (B103, DHSB, 0.5 mg/mL), anti-FASN (10624-2-AP, Proteintech, 1:200), anti-CPT1 (bs-23779R, Bioss, 1:500), anti-ULK1 (bs-3602R, Bioss,1:500), anti-LC3B (NB100-2220, Novus, 2.0 mg/mL), anti-P62 (18420-1-AP, Proteintech, 1:1,000), anti-TBP (44059, Cell signaling, 1:1000), anti-KLF4 (bs-1064R, Bioss,1:500), anti-GPI (GTX113203, GeneTex, 1:500), anti-TNNI2 (bs-10617R, Bioss, 1:500), anti-CDKN1A (GTX112898, GeneTex, 1:500), and anti-GAPDH (60004-1-Ig, Proteintech, 1:5,000). ProteinFind goat anti-mouse IgG(H + L), HRP conjugate (HS201-01, TransGen, 1:1,000) and ProteinFind goat anti-rabbit IgG(H + L), HRP conjugate (HS101-01, TransGen, 1:500) were used as secondary antibodies. The original images of western blot are shown in Additional file 1.

### RNA pull-down assay and RIP assay

Biotinylated RNAs were harvested by using a Ribo RNAmax-T7 biotin-labeled transcription kit (RiboBio, China). A Pierce Magnetic RNA–Protein Pull-Down Kit (Thermo Fisher Scientific) was used in RNA–protein pull-down experiments according to the manufacturer’s instructions. The eluted products were identified by mass spectrometry with a Q Exactive mass spectrometer (Thermo Fisher) or western blot.

Immunoprecipitation Kit (Millipore, USA) according to the manufacturer’s instructions. The antibody used for RIP assays was anti-TBP (44059, Cell Signaling, 1:100).

### Chromatin immunoprecipitation (CHIP)

TBP ChIP was performed with ChIP Kit (Millipore, Bedford, MA) according to the manufacturer’s instructions. Briefly, the ChIPed DNA was eluted, reverse X-linked, purified, and analyzed by qRT-PCR. All primers used in ChIP-qPCR are presented in Additional file 10: Table S4.

### Luciferase reporter assay

For luciferase reporter assay, reporter plasmids with the promoter region of *KLF4*, *GPI*, *TNNI2*, and *CDKN1A* were transfected into CPMs by Lipofectamine 3000 (Invitrogen, USA) in 96-well plates. The luciferase activities were measured 48 h after differentiation by using Dual-Luciferase®Reporter Assay System (Promega, Madison, WI, USA). Firefly activity was normalized to Renilla luciferase activity.

### Statistical analysis

In this study, all experiments were repeated at least three times, and results were represented as mean ± SEM. Where applicable, the statistical significance of the data was tested using independent sample *t*-test or ANOVA followed by Dunnett’s test. The types of tests and the *P*-values, when applicable, are indicated in the figure legends.

## Results

### *LncRNA-TBP* is a novel lncRNA associated with myogenesis

**Fig. 1:**
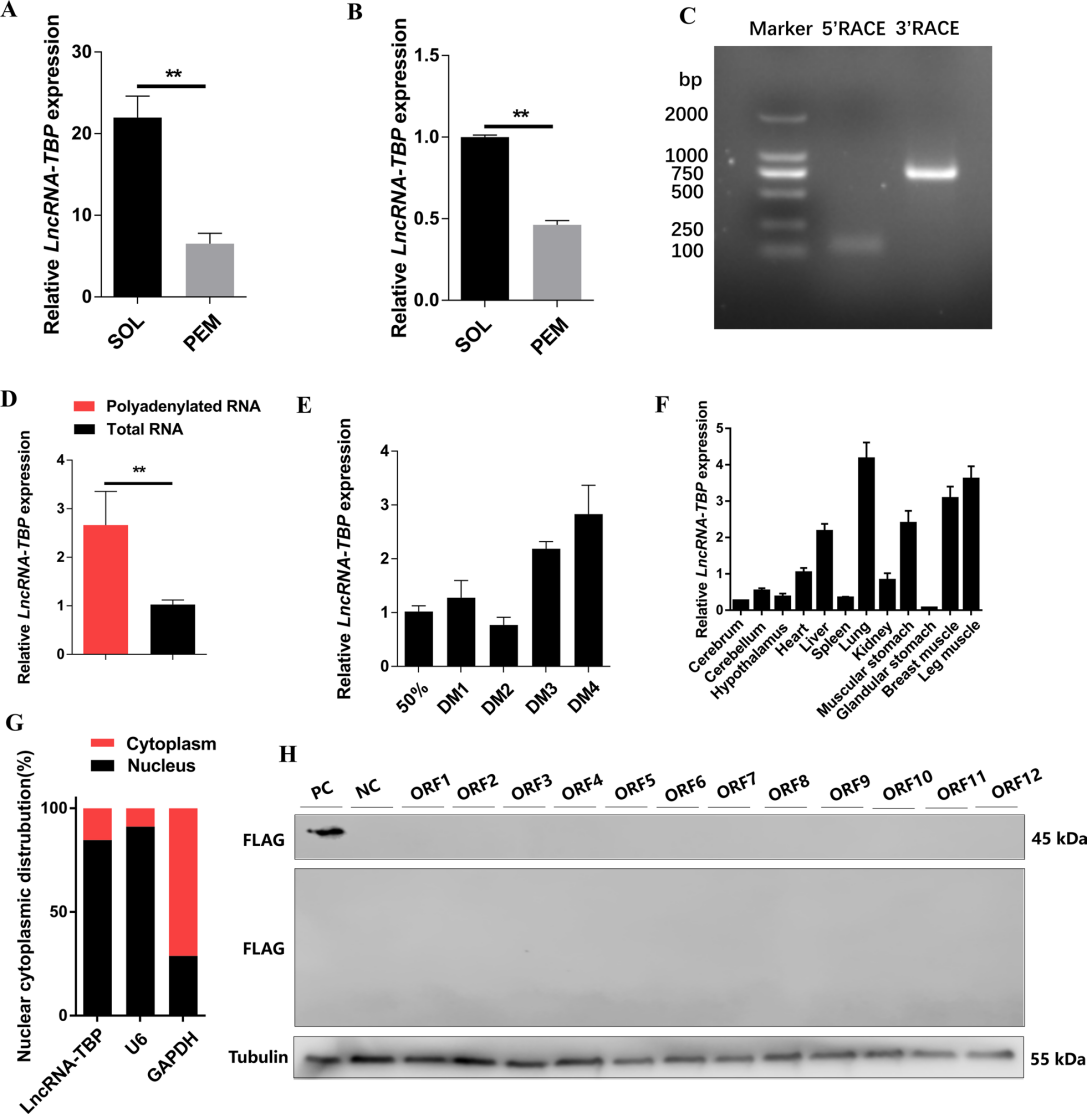
Identification of *LncRNA-TBP*. **A** *LncRNA-TBP* expression of pectoralis major and soleus of 7-week-old Xinghua chickens by RNA-seq (n = 4). **B** Relative *LncRNA-TBP* expression of pectoralis major and soleus of 7-week-old Xinghua chickens by qRT-PCR (n = 4). **C** Results of *LncRNA-TBP* 5’ RACE and 3’ RACE. **D** Relative *LncRNA-TBP* expression in polyadenylated RNA and total RNA (n = 4). **E** Relative *LncRNA-TBP* expression during CPMs proliferation and differentiation (n = 3). **F** Tissues expression profiles of *LncRNA-TBP*. The horizontal axis and vertical axis indicate different tissues and their relative expression values, respectively (n = 3). **G** The distribution of *LncRNA-TBP* in the cytoplasm and nuclei of CPMs was determined by qRT-PCR. GAPDH and U6 serve as cytoplasmic and nuclear localization controls, respectively. **H** Western blot analysis of the coding ability of *LncRNA-TBP*. The potential ORFs of *LncRNA-TBP* were cloned into the pcDNA3.1-3xFlag-C vector. CPMs transfected with β-actin were used as a positive control (PC) and untransfected CPMs were used as a negative control (NC). Results are presented as mean ± SEM. In panels (**A**, **B** and **D**), the statistical significance of differences between means was assessed using independent sample *t*-test. (*P < 0.05; **P < 0.01)

Our previous RNA-seq study found a muscle-related lncRNA (*LncRNA-TBP*) was highly expressed in SOL (Fig. 1A, B). 5′ and 3′ ends of *LncRNA-TBP* were identified by RACE analysis (Fig. 1C). The NCBI BLAST indicated that *LncRNA-TBP* located on Chromosome 3 and spanned from 82341588 to 82342330, and 82369736 to 82370049 with 1057 nt long, relatively conserved in *Meleagris gallopavo*, *Apteryx mantelli mantelli*, and *Numida meleagris* (Additional file 2: Fig. S1 and Additional file 10: Table S1). *LncRNA-TBP* highly expressed in polyadenylated RNA (Fig. 1D). *LncRNA-TBP* upregulated during myogenic differentiation, and enriched in leg muscles and breast muscles (Fig. 1E, F), implying that it may play an important role in skeletal muscle development. In addition, cell-fractionation assays demonstrated that *LncRNA-TBP* is mainly present in the nucleus of chicken primary myoblasts (CPMs) (Fig. 1G). To further prove the coding potential of *LncRNA-TBP*, we analyzed the twelve potential ORFs of *LncRNA-TBP* by western blot. The results show that *LncRNA-TBP* is a lncRNA without protein-encoding potential (Fig. 1H).

### *LncRNA-TBP* inhibits myoblast proliferation and promotes myoblast differentiation

**Fig. 2:**
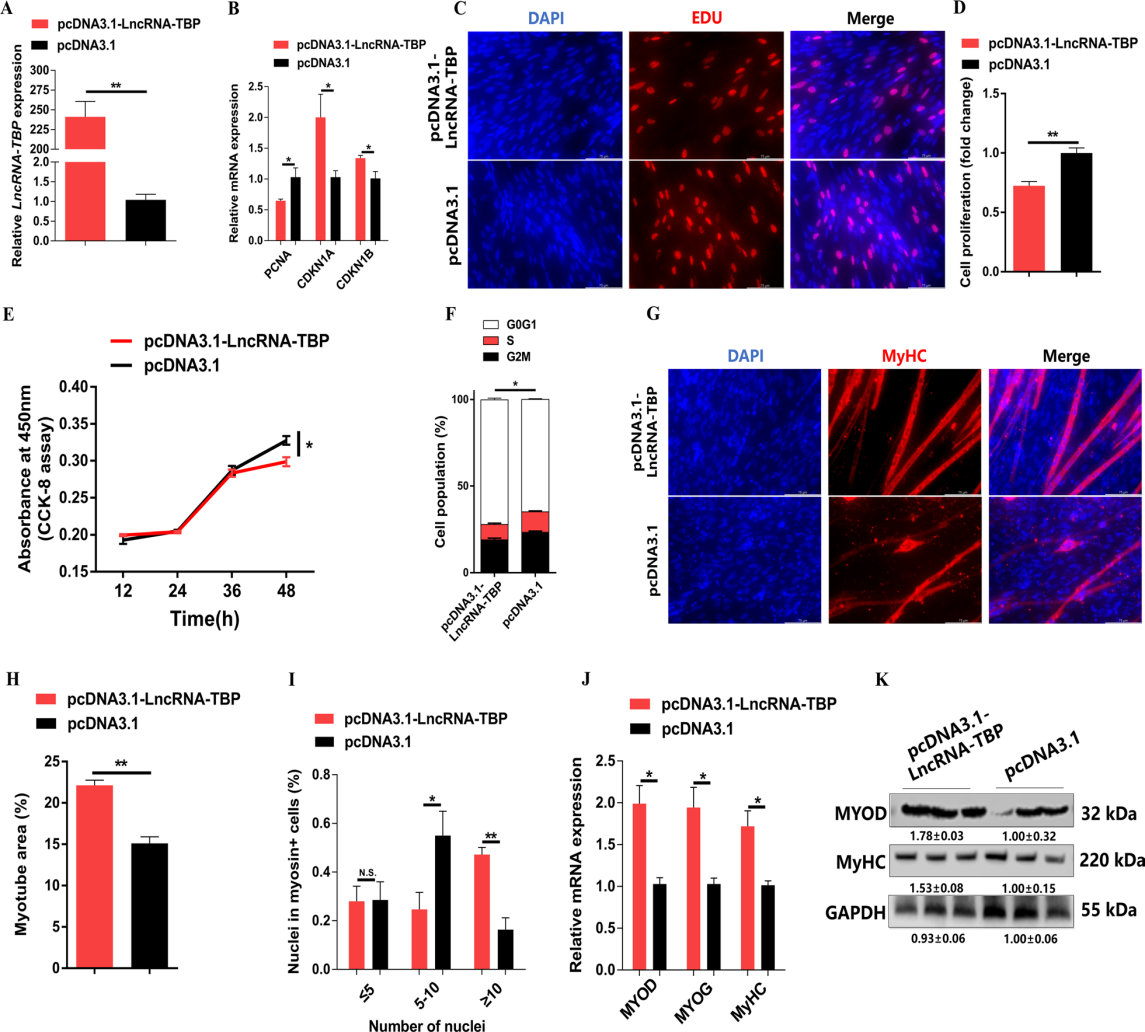
*LncRNA-TBP* inhibits myoblast proliferation but promotes myogenic differentiation. **A** Relative *LncRNA-TBP* expression with *LncRNA-TBP* overexpression in vitro (n = 6). **B** Relative mRNA levels of several cell cycle genes with overexpression of *LncRNA-TBP* (n = 6). **C** The proliferation of transfected CPMs was assessed by 5-ethynyl-2’-deoxyuridine (EdU) incorporation (n = 3). **D** The proliferation rate of myoblasts after the overexpression of *LncRNA-TBP* (n = 8). **E** CCK-8 assays were performed in CPMs with *LncRNA-TBP* overexpression (n = 6). **F** Cell cycle analysis of myoblasts after the overexpression of *LncRNA-TBP* (n = 4). **G–I** MyHC immunostaining (n = 3) (**G**), myotube area (%) (n = 8) (**H**) and myoblast fusion index (n = 8) (**I**) of CPMs transduced with *LncRNA-TBP* overexpression. Cells were differentiated for 72 h after transfection. **J** and **K** Relative mRNA (n = 6) (**J**) and protein (n = 3) (**K**) expression levels of myoblast differentiation marker genes with *LncRNA-TBP* overexpression. In panel **H**, the numbers shown below the bands were folds of band intensities relative to control. Band intensities were quantified by ImageJ and normalized to GAPDH. Data are expressed as a fold-change relative to the control. Results are shown as mean ± SEM. In panels (**A**, **B**, **D**–**F** and **H–J**), the statistical significance of differences between means was assessed using an independent sample *t*-test. (*P < 0.05; **P < 0.01; N.S., no significant difference)

*LncRNA-TBP* was predominantly expressed in breast muscle and leg muscle (Fig. 1F), implying that *LncRNA-TBP* plays an important role in myogenesis. To assess the effect of *LncRNA-TBP* on proliferation and differentiation of myoblast, the overexpression vector and inhibitor of *LncRNA-TBP* were transfected into CPMs (Fig. 2A and Additional file 3: Fig. S2A). Overexpression of *LncRNA-TBP* increased the expression of cell cycle-inhibiting genes like *CDKN1A* and *CDKN1B* while decreasing the expression level of cell cycle-promoting genes like *PCNA*. The opposite result was observed with *LncRNA-TBP* knockdown (Fig. 2B and Additional file 3: Fig. S2B). The 5-ethynyl-2’-deoxyuridine (EdU) staining and cell counting kit-8 (CCK-8) assay demonstrated that *LncRNA-TBP* overexpression significantly inhibited myoblast proliferation and viability (Fig. 2C–E). Conversely, interference with *LncRNA-TBP* promoted EdU incorporation and myoblast proliferation (Additional file 3: Fig. S2C–E). At the same time, overexpression of *LncRNA-TBP* significantly increased the number of G0/G1 cells, and the number of S phase cells was lower than the control group, whereas myoblast division was inhibited with *LncRNA-TBP* interference (Fig. 2F and Additional file 3: Fig. S2F).

To further investigate the potential function of *LncRNA-TBP* in myoblast differentiation, immunofluorescence staining was performed after overexpression and inhibition of *LncRNA-TBP.* The results showed that overexpression of *LncRNA-TBP* increased the total areas of myotubes, while myotube formation was facilitated (Fig. 2G–I). In contrast, *LncRNA-TBP* interference suppressed myoblast differentiation (Additional file 3: Fig. S2G–I). Moreover, the expressions level of myoblast differentiation marker genes, including *MYOD*, *MYOG*, and *MyHC* were significantly upregulated with *LncRNA-TBP* overexpression (Fig. 2J, K). Conversely, *LncRNA-TBP* interference repressed their expression (Additional file 3: Fig. S2J, K).

### *LncRNA-TBP* accelerates fatty acid oxidation, and enhances TCA cycle flux in skeletal muscle

**Fig. 3:**
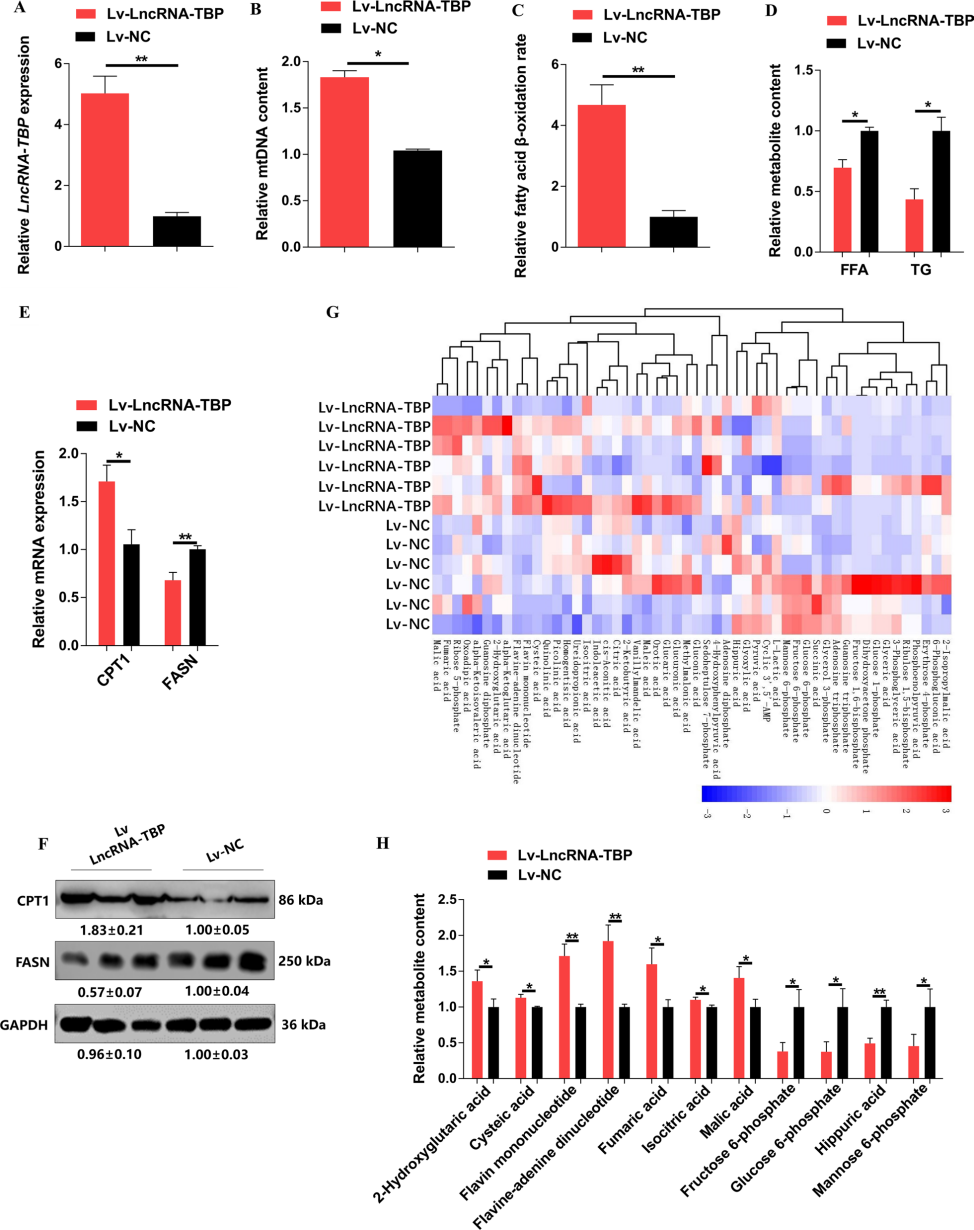
*LncRNA-TBP* promotes fatty acid oxidation and TCA cycles in skeletal muscle. **A** Relative *LncRNA-TBP* expression in gastrocnemius after infected with lentivirus-mediated *LncRNA-TBP* overexpression (Lv-LncRNA-TBP) or negative control (Lv-NC) (n = 4). **B** Relative mtDNA content in *LncRNA-TBP* overexpression gastrocnemius (n = 4). **C** Relative fatty acid β-oxidation rate with overexpression of *LncRNA-TBP* in gastrocnemius (n = 4). **D** Relative free fatty acid (FFA) and triglyceride (TG) content in gastrocnemius with *LncRNA-TBP* overexpression (n = 4). **E** and **F** Relative mRNA (n = 6) (**E**) and protein (n = 3) (**F**) expression levels of fatty acid oxidation or synthesis related-genes in gastrocnemius with *LncRNA-TBP* overexpression. **G** Hierarchical clustering analysis (HCA) of metabolites in gastrocnemius with overexpression of *LncRNA-TBP* (n = 6). The colors indicate the relative levels in the overexpression of *LncRNA-TBP* or control group. **H** Relative metabolite content of glycolysis and tricarboxylic acid (TCA) cycle in gastrocnemius with *LncRNA-TBP* overexpression (n = 6). In panel **F**, the numbers shown below the bands were folds of band intensities relative to control. Band intensities were quantified by ImageJ and normalized to GAPDH. Data are expressed as a fold-change relative to the control. Results are shown as mean ± SEM. In panels (**A–E** and **H**), the statistical significance of differences between means was assessed using an independent sample *t*-test. (*P < 0.05; **P < 0.01; N.S., no significant difference)

To verify whether *LncRNA-TBP* regulates skeletal muscle development in vivo, lentiviral-mediated *LncRNA-TBP* overexpression (LV-LncRNA-TBP) or cholesterol-modified antisense oligonucleotide (Chol-ASO-LncRNA-TBP) were injected to the gastrocnemius of Xinghua chicken (Fig. 3A and Additional file 4: Fig. S3A). *LncRNA-TBP* overexpressed increased mitochondrial DNA content, which potentially contributed to the acceleration of fatty acid oxidation (FAO) and inhibited the accumulation of free fatty acid (FFA) and triglyceride (TG) (Fig. 3B–D). In contrast, mitochondrial DNA content and fatty acid β-oxidation were reduced after the *LncRNA-TBP* knockdown (Additional file 4: Fig. S3B–D). Besides, the qPCR and western blotting analyses showed that knockdown of *LncRNA-TBP* downregulated FAO-related genes like *CPT1* and upregulating key genes involved in fatty acid synthesis (such as *FASN*), while opposite results were shown with *LncRNA-TBP* overexpression (Fig. 3E, F and Additional file 4: Fig. S3E, F).

Mitochondria switch between lipid and glucose oxidation through the TCA cycle to generate ATP, which is pivotal for maintaining systemic energy homeostasis [19–22]. Given that overexpression of *LncRNA-TBP* promoted the content of mitochondrial DNA (Fig. 3B), we performed a comparative metabolome analysis to study whether *LncRNA-TBP* functions muscle metabolism. The result of hierarchical clustering analysis (HCA) separated controls and overexpression of *LncRNA-TBP* (Fig. 3G and Additional file 11: Table S2). For example, compared with control, glycolytic metabolites such as fructose 6-phosphate and glucose 6-phosphate were significantly decreased with *LncRNA-TBP* overexpression (Fig. 3H and Additional file 11: Table S2). In the meantime, metabolites of the TCA cycle, including malic acid, isocitric acid, and fumaric acid were significantly promoted (Fig. 3H and Additional file 11: Table S2). Altogether, our results indicated that *LncRNA-TBP* decreases the end products of glycolysis and elevates metabolites of the TCA cycle by promoting mitochondrial function, leading to reduction of lipid accumulation.

### *LncRNA-TBP* activates slow-twitch muscle phenotype and induces muscle hypertrophy

**Fig. 4:**
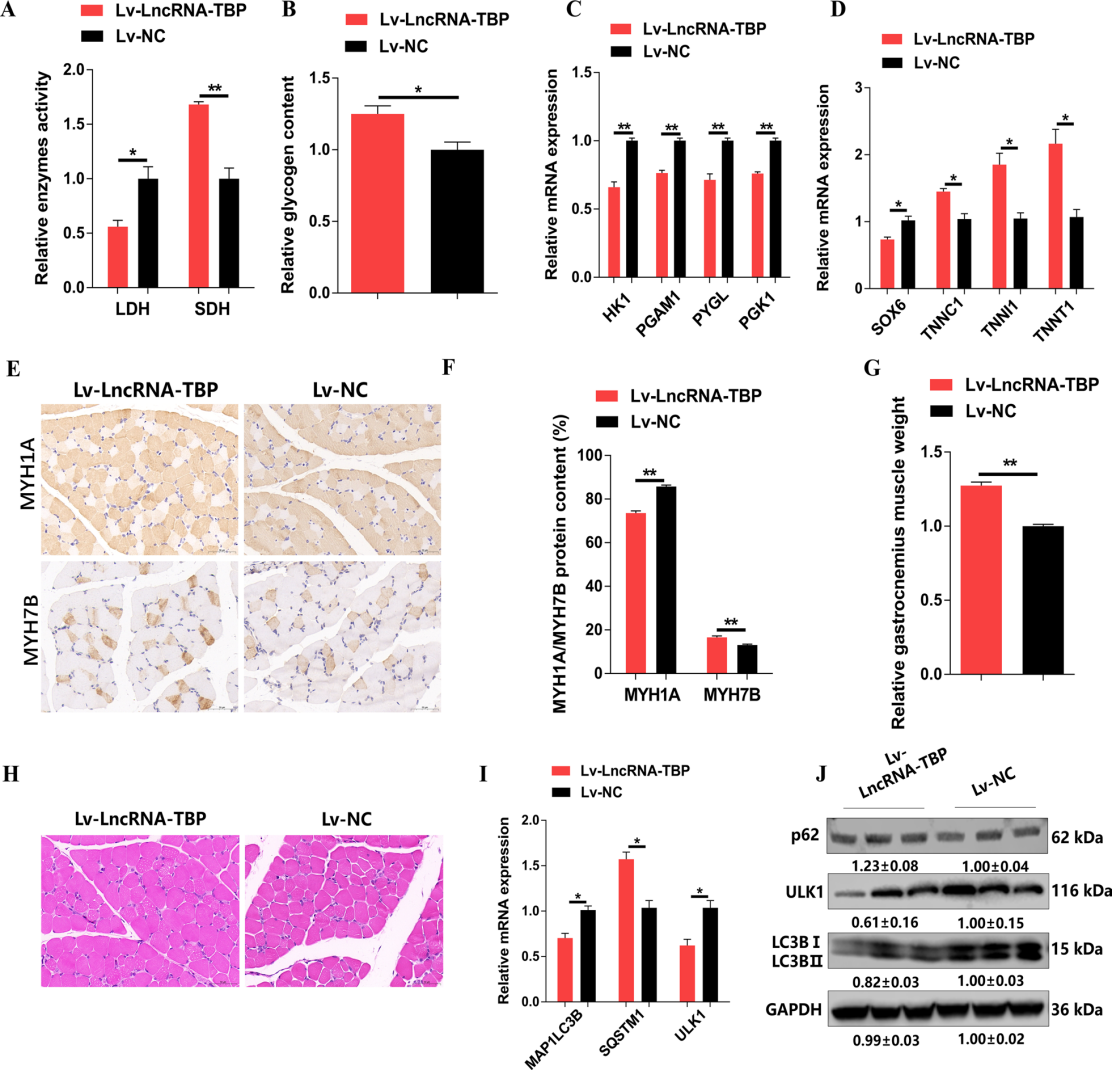
*LncRNA-TBP* activates slow-twitch muscle phenotype and induce muscle hypertrophy. **A** Relative enzymes activity of lactic dehydrogenase (LDH) and succinate dehydrogenase (SDH) in gastrocnemius infected with *LncRNA-TBP* overexpression (n = 4). **B** Relative glycogen content in *LncRNA-TBP* overexpression gastrocnemius (n = 5). **C** Relative mRNA expression levels of glycogenolytic and glycolytic genes in *LncRNA-TBP* overexpression gastrocnemius (n = 6). **D** Relative mRNA expression levels of several fast-/slow-twitch myofiber genes in overexpression of *LncRNA-TBP* gastrocnemius (n = 6). **E** and **F** Immunohistochemistry analysis of MYH1A/MYH7B (n = 3) (**E**) and MYH1A/MYH7B protein content (n = 8) (**F**) in gastrocnemius with *LncRNA-TBP* overexpression. **G** Relative gastrocnemius muscle weight after infected with lentivirus-mediated *LncRNA-TBP* overexpression (n = 6). **H** H&E staining in overexpression of *LncRNA-TBP* gastrocnemius (n = 3). **I** and **J** Relative mRNA (n = 6) (**I**) and the protein (n = 3) (**J**) expression levels of the autophagy-related genes in gastrocnemius with *LncRNA-TBP* overexpression. In panel **J**, the numbers shown below the bands were folds of band intensities relative to control. Band intensities were quantified by ImageJ and normalized to GAPDH. Data are expressed as a fold-change relative to the control. Results are shown as mean ± SEM. In panels **A**–**D**, **F** and **H–I**, the statistical significance of differences between means was assessed using an independent sample *t*-test. (*P < 0.05; **P < 0.01; N.S., no significant difference)

Skeletal muscle development is primarily regulated by fiber type composition and muscle fiber size. The composition of myofiber types is closely related to the way muscles are metabolized [23, 24]. Given that *LncRNA-TBP* is highly expressed in SOL and mediated the flux of glycolysis and TCA cycle, we further examined whether *LncRNA-TBP* could affect the conversion of skeletal muscle fiber types in vivo. As expected, the activity of lactate dehydrogenase (LDH) was suppressed, while the activity of succinate dehydrogenase (SDH) was enhanced with *LncRNA-TBP* overexpression (Fig. 4A). Meanwhile, glycogen content was increased and expression of glycogenolytic and glycolytic genes was downregulated with overexpression of *LncRNA-TBP* (Fig. 4B, C). The opposite results were shown with the knockdown of *LncRNA-TBP* (Additional file 5: Fig. S4A-C). The expression levels of fast-twitch myofiber genes like *SOX6* and slow-twitch myofiber genes (such as *TNNC1*, *TNNI1* and *TNNT1*) were further tested. It was found that overexpression of *LncRNA-TBP* promoted expressions of slow-twitch myofiber genes (Fig. 4D). More importantly, results of immunohistochemistry showed that *LncRNA-TBP* overexpression promoted the expression level of MYH7B/slow-twitch protein and suppressed the expression level of MYH1A/fast-twitch protein (Fig. 4E, F). On the contrary, *LncRNA-TBP* knockdown upregulated the fast-twitch protein level and drove the transformation of slow-twitch to fast-twitch myofibers (Additional file 5: Fig. S4D-F).

Recent evidences have revealed that remodeling of skeletal muscle fiber types can affect muscle mass, and induce muscle hypertrophy and muscle atrophy by anabolic and catabolic signaling pathways, respectively [25]. *LncRNA-TBP* overexpression leads to increased muscle mass and cross-sectional area (CSA), while the opposite result occurred upon *LncRNA-TBP* knockdown (Fig. 3G and H and Additional file 5: Fig. S4G, H), suggesting that *LncRNA-TBP* regulates skeletal muscle hypertrophy. Autophagy is a highly conserved homeostatic process carrying out degradation of cytoplasmic components including damaged organelles, toxic protein aggregates, and intracellular pathogen [26]. Maintaining basal autophagy flux is essential to clear damaged organelles or recycle macromolecules in muscles during metabolic stress [27]. To further explore the regulatory mechanism of *LncRNA-TBP* in inducing muscle hypertrophy, we detected expressions of autophagy-related genes. *LncRNA-TBP* overexpression upregulated the expression level of *SQSTM1*, whereas expressions of autophagy-related genes (such as *MAP1LC3B*, and *ULK1*) and content of LC3BII were downregulated (Fig. 4I, J). Conversely, *LncRNA-TBP* knockdown activated autophagy (Additional file 5: Fig. S4I, J), suggesting that *LncRNA-TBP* may promote muscle hypertrophy by decreasing basal autophagy flux.

### *LncRNA-TBP* directly interacts with TBP

**Fig. 5:**
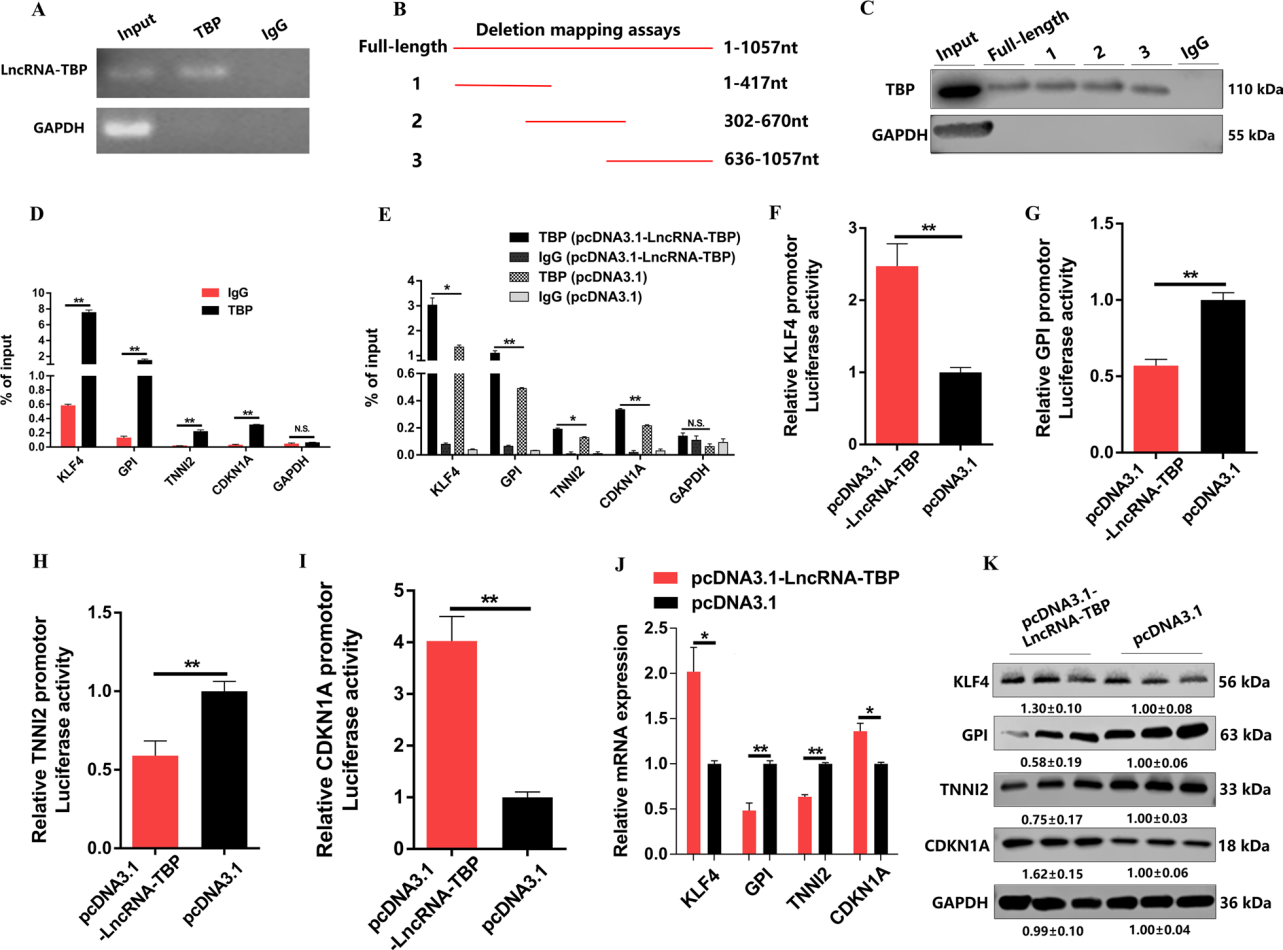
*LncRNA-TBP* interacts with TBP to regulate the transcriptional activity of TBP-target genes. **A** *LncRNA-TBP* interacts with TBP protein were determined by RNA Immunoprecipitation (RIP). **B** and **C** The interaction of full-length and truncated *LncRNA-TBP* (base pairs 1-417, 302-670 and 636-1057) (**B**) with TBP protein was determined by RNA pulldown (**C**). **D** ChIP-qPCR results showing that TBP enrichment at the *KLF4*, *GPI*, *TNNI2*, and *CDLN1A* promoters in CPMs (n = 3). **E** ChIP-qPCR results showing that *LncRNA-TBP* overexpression significantly increased TBP enrichment at the *KLF4*, *GPI*, *TNNI2*, and *CDLN1A* promoters in CPMs (n = 3). **F**–**I** The luciferase activities of the reporter show that the relative promoter activity of *KLF4* (**F**), *GPI* (**G**), *TNNI2* (**H**), and *CDKN1A* (**I**) significantly changed with *LncRNA-TBP* overexpression in CPMs (n = 6). **J–K** The relative mRNA (n = 4) (**J**) and protein (n = 3) (**K**) expression levels of *KLF4*, *GPI*, *TNNI2*, and *CDKN1A* after *LncRNA-TBP* overexpression. In panels **A**, **C**, and **K**, the numbers shown below the bands were folds of band intensities relative to control. Band intensities were quantified by ImageJ and normalized to β-Tubulin. Data are expressed as a fold-change relative to the control. Results are presented as mean ± SEM. In panels **D**–**J**, the statistical significance of differences between means was assessed using an independent sample *t*-test. (*P < 0.05; **P < 0.01; N.S., no significant difference)

Recent studies have found that many nuclear lncRNAs perform their functions through interaction with proteins [28]. The nuclear localization of *LncRNA-TBP* suggested that this lncRNA may modulates the transcriptional regulation of target genes. Thus, we attempted to identify the protein partners of *LncRNA-TBP*. First, the potential *LncRNA-TBP*-binding proteins were predicted using the RNA–protein interaction prediction (RPISeq), and TBP was found may interact with *LncRNA-TBP* (Additional file 6: Fig. S5A). To validate this interaction between *LncRNA-TBP* and TBP, we performed a RNA immunoprecipitation (RIP) analysis in CPMs. As expected, reverse transcription-polymerase chain reaction (RT-PCR) analysis of antibody-enriched RNA revealed that TBP antibody pulled down significantly more *LncRNA-TBP* than the IgG control (Fig. 5A), suggesting that TBP interacts with *LncRNA-TBP*. To determine the core protein-binding domain of *LncRNA-TBP*, we constructed a series of truncated *LncRNA-TBP* fragments. We found that like full-length *LncRNA-TBP*, all of the truncated fragments could physically bind TBP (Fig. 5B, C). Collectively, these findings showed that *LncRNA-TBP* directly interacts with TBP.

### *LncRNA-TBP* regulates transcriptional activity of TBP-target genes by binding to TBP

The general transcription factor (TBP) is a key initiation factor involved in transcription by all three eukaryotic RNA polymerases, is required for every single transcription event in eukaryotes [29–32]. Through our previous ATAC sequencing analysis, a total of 20 target genes (e.g., glycolysis-related genes (*GPI*), cell proliferation-related genes (*CDKN1A* and *KLF4*) and fast muscle-related genes (*TNNI2*)) were predicted be regulated by TBP (Additional file 6: Fig. S5B and Additional file 12: f S3). Gene ontology (GO) and Kyoto Encyclopedia of Genes and Genomes (KEGG) enrichment analysis found that these TBP-target genes were mainly enriched in biological processes such as cellular process, metabolic process, cellular component organization or biogenesis, and biological regulation, as well as participated in biological processes including metabolic pathways, carbon metabolism and so on (Additional file 6: Fig. S5C, D). By performing ChIP-qPCR, we validated that TBP can bind and regulate the promoter of *KLF4*, *GPI*, *TNNI2*, and *CDKN1A* (Fig. 5D).

Because *LncRNA-TBP* specifically interacts with TBP, we investigated whether there is a mutual regulation relationship between *LncRNA-TBP* and TBP in CPMs. The results showed that *LncRNA-TBP* knockdown or overexpression did not significantly influence TBP mRNA and protein expression (Additional file 7: Fig. S6A–D). These results suggested that *LncRNA-TBP* may regulate myogenesis through its interaction with TBP rather than by regulating TBP gene expression. Given that TBP functions in regulate the promoter activity of its target genes, we also performed ChIP-qPCR to elucidate whether *LncRNA-TBP* affects the capacity of TBP to bind the promoters of its target genes. *LncRNA-TBP* overexpression significantly increased the enrichment of TBP to the promoter of *KLF4*, *GPI*, *TNNI2*, and *CDKN1A,* whereas the results were reversed after *LncRNA-TBP* knockdown (Fig. 5E and Additional file 8: Fig. S7A). Next, to determine whether *LncRNA-TBP* regulates promoter activity of TBP-target genes such as *KLF4*, *GPI*, *TNNI2*, and *CDKN1A*, luciferase reporter assays were performed. Overexpression of *LncRNA-TBP* promoted the promoter activity of *KLF4* and *CDKN1A* while inhibiting the promoter activity of *GPI* and *TNNI2* (Fig. 5F–I). Consistently, the knockdown of *LncRNA-TBP* had opposite effects in CPMs (Additional file 8: Fig. S7B-E). We further examined the mRNA and protein expressions of *KLF4*, *GPI*, *TNNI2*, and *CDKN1A*. As expected, *LncRNA-TBP* would promote the expression of *KLF4* and *CDKN1A*, while decreasing the expression of *GPI* and *TNNI2* (Fig. 5J, K and Additional file 8: Fig. S7F, G), suggesting that *LncRNA-TBP* can modulate transcriptional activity of TBP-target genes by binding to TBP protein.

### *TBP* is involved in myogenesis

**Fig. 6:**
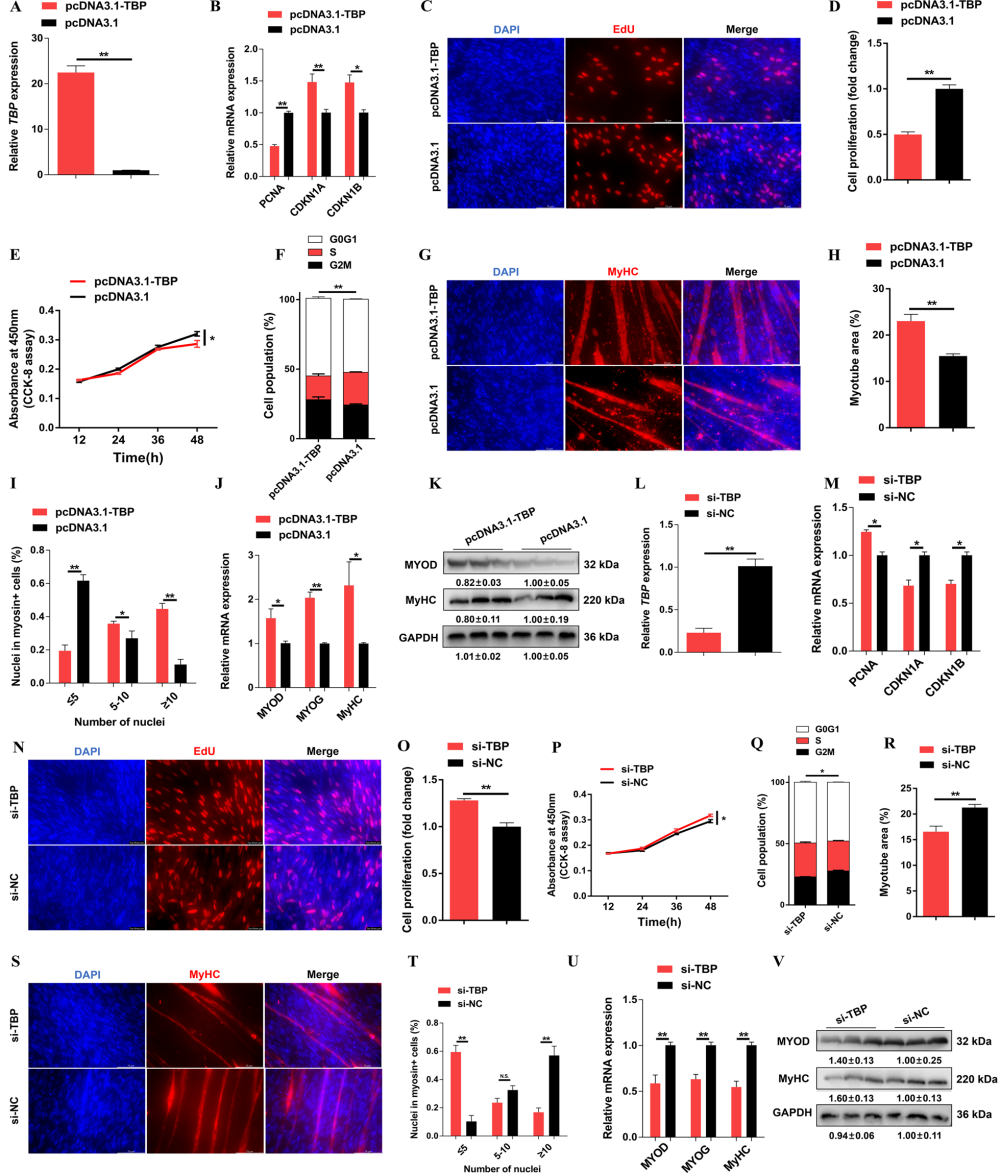
*TBP* inhibits myoblast proliferation and promotes myogenic differentiation. **A** Relative *TBP* expression with *TBP* overexpression in vitro (n = 6). **B** Relative mRNA levels of several cell cycle genes with overexpression of *TBP* (n = 4). **C** The proliferation of transfected CPMs was assessed by 5-ethynyl-2’-deoxyuridine (EdU) incorporation (n = 3). **D** The proliferation rate of myoblasts after the overexpression of *TBP* (n = 8). **E** CCK-8 assays were performed in CPMs with *TBP* overexpression (n = 6). **F** Cell cycle analysis of myoblasts after the overexpression of *TBP* (n = 4). **G**–I MyHC immunostaining (n = 3) (**G**), myotube area (%) (n = 8) (**H**) and myoblast fusion index (n = 8) (**I**) of CPMs transduced with *TBP* overexpression. Cells were differentiated for 72 h after transfection. **J** and **K** Relative mRNA (n = 6) (**J**) and protein (n = 3) (**K**) expression levels of myoblast differentiation marker genes with *TBP* overexpression. **L** Relative *TBP* expression with *TBP* interference in vitro (n = 3). **M** Relative mRNA levels of several cell cycle genes with interference of *TBP* (n = 4). **N** The proliferation rate of myoblasts after the interference of *TBP* (n = 8). **O** The proliferation of transfected CPMs was assessed by 5-ethynyl-2’-deoxyuridine (EdU) incorporation (n = 3). **P** CCK-8 assays were performed in CPMs with *TBP* interference (n = 6). **Q** Cell cycle analysis of myoblasts after the interference of *TBP* (n = 4). **R–T** yotube area (%) (n = 8) (**R**), MyHC immunostaining (n = 3) (**S**) and myoblast fusion index (n = 8) (**T**) of CPMs transduced with *TBP* interference. Cells were differentiated for 72 h after transfection. **U** and **V** Relative mRNA (n = 6) (**U**) and protein (n = 3) (**V**) expression levels of myoblast differentiation marker genes with *TBP* interference. In panels **K** and **V**, the numbers shown below the bands were folds of band intensities relative to control. Band intensities were quantified by ImageJ and normalized to GAPDH. Data are expressed as a fold-change relative to the control. Results are shown as mean ± SEM. In panels **A**, **B**, **D**–**F, H–J, L–N, O–R** and **T**, **U**), the statistical significance of differences between means was assessed using an independent sample *t*-test. (*P < 0.05; **P < 0.01; N.S., no significant difference)

TBP was upregulated during myoblast differentiation (Additional file 9: Fig. S8A), implying that it may play an important role in skeletal muscle development. Moreover, Subcellular location annotation showed that TBP protein exists in the nucleus (Additional file 9: Fig. S8B). To explore the potential biological functions of *TBP* in myogenesis, we examined the effects of *TBP* in myoblasts proliferation and differentiation in vivo. *TBP* was successfully overexpressed or knockdown in CPMs (Fig. 6A, L). Overexpression of *TBP* reduced cell-cycle-promoting genes while increasing the expression of cell-cycle-inhibiting genes. The EdU and CCK-8 assays showed that overexpression of *TBP* decreased EdU incorporation and repressed myoblast viability, whereas its inhibition promoted myoblast proliferation (Fig. 6B–E, N–P). Moreover, flow cytometric analysis revealed that *TBP* overexpression reduced the number of S-phase cells (Fig. 6F). Conversely, *TBP* inhibition resulted in a greater number of S-phase cells (Fig. 6Q).

Next, immunofluorescence staining was performed to detected the role of *TBP* in myogenetic differentiation. *TBP* overexpression significantly facilitated myoblast differentiation, increased the total areas of myotubes and myotube formation (Fig. 6G–I). Meanwhile, qPCR and western blotting showed that expressions of myoblast differentiation marker genes were upregulated with *TBP* overexpression (Fig. 6J, K). In contrast, *TBP* interference repressed myoblast differentiation (Fig. 6R–V). Taken together, these data indicated that *TBP* suppresses myoblast proliferation and promotes myoblast differentiation, which is similar to *LncRNA-TBP* in function.

## Discussion

Myogenesis is a highly ordered process including myoblast proliferation and differentiation, myotube formation and maturity, is controlled by a series of myogenic regulatory factors [33]. Many studies have suggested the important role of lncRNAs in skeletal muscle myogenesis while highlighting the necessity to systematically identify lncRNAs altered in skeletal muscle development [20, 34, 35]. The composition of myofiber types may influence meat quality by affecting the content of metabolites postmortem in livestock such as pH, meat color, and drip loss [36, 37]. Compare to the fast-twitch muscle phenotype, the proportion of type slow-muscle fibers is proportional to the content of intramuscular fat, which have higher tenderness, flavor, and juiciness [38, 39]. Here, we found that *LncRNA-TBP* is highly expressed in skeletal muscle, and its expression gradually increased with the stage of myoblast differentiation. Functional analyses showed that *LncRNA-TBP* suppresses myoblast proliferation and induces myogenic differentiation in vivo, *LncRNA-TBP* activated the slow-twitch muscle phenotype and reduces fat deposition.

Skeletal muscle can maintain systemic energy homeostasis in response to various metabolic stresses through regulating glucose uptake, lipid storage, and energy balance [40]. In this study, we found that *LncRNA-TBP* increases cellular mitochondrial DNA content and facilitates fatty acid oxidation in skeletal muscle, resulting in inhibiting the deposition of intramuscular fat. In the meantime, *LncRNA-TBP* reduced glycolytic capacity and increase oxidative capacity of skeletal muscle, which suppressed the autophagy pathway and reduced muscle atrophy.

TATA-binding protein (TBP) is a key component of the general transcription machinery that is involved in transcription by all three eukaryotic RNA polymerases [27–30]. Recent studies have found that mutant of TBP decreased its association with *MyoD*, which is a muscle-specific transcription factor, and caused a dramatic shrink in skeletal muscle mass [41, 42]. As a TATA-binding protein (TBP) associated factors, TAF9b was found can act a coactivator to stabilize the structure of P53 and promote P53 activation, thus reducing glycolysis, increasing superoxide levels, and inhibiting autophagy [12–15]. Notably, recent evidences have revealed that LncRNAs are able to be widely involved in a variety of biological processes through recruit RNA-binding proteins to regulate the transcription of target genes [43–45]. Here, we found *LncRNA-TBP* is a novel player in TBP-regulating network that can regulate the transcriptional activity of TBP-target genes by recruiting TBP protein, thus modulating myogenesis progression and inducing slow-twitch fibers.

## Conclusion

**Fig. 7:**
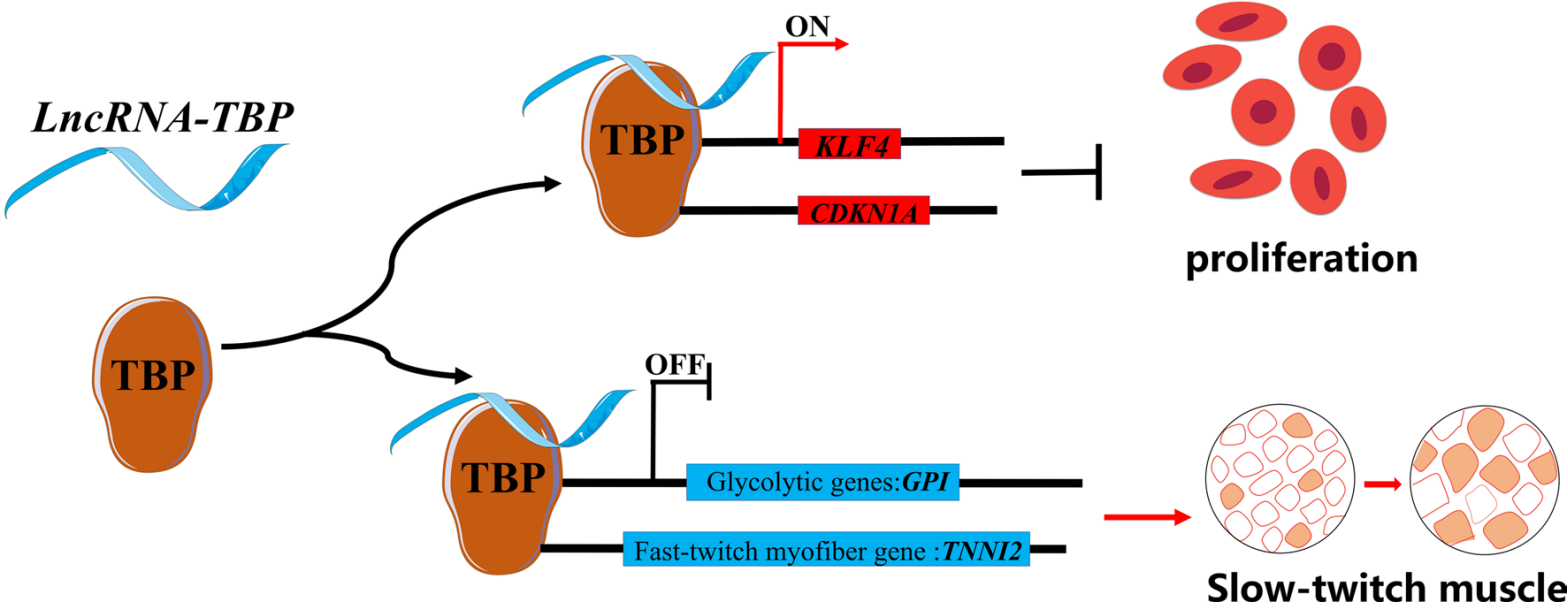
Model of *LncRNA-TBP* interacts with TBP to regulate the transcriptional activity of TBP-target genes, thus inhibiting myoblast proliferation, as well as activating slow-twitch muscle phenotype and inducing muscle hypertrophy

In conclusion, we identify an lncRNA, *LncRNA-TBP*, and propose a mechanistic model to elucidate its role in the regulation of myogenesis and myofiber transformation through TBP-mediated transcriptional regulation (Fig. 7). Our findings present a novel model about the regulation of lncRNA in myogenesis, and will contribute to the development of further research.

## Supplementary Information

**Additional file 1.** Original data of WB.

**Additional file 2: Figure S1**. Conservative analysis of *LncRNA-TBP* performed by using the NCBI’s BLAST. A total of eighteen species, including Anas platyrhynchos, Anser cygnoides, Apteryx mantelli mantelli, Aquila chrysaetos, Bos taurus, Coturnix japonica, Gallus gallus, Geospiza fortis, Homo sapiens, Meleagris gallopavo, Melopsittacus undulatus, Mus musculus, Numida meleagris, Ovis aries, Pan troglodytes, Rattus norvegicus, Sus scrofa and Zebra finch were used for Nucleotide BLAST. Top 4 most conservative results were listed above.

**Additional file 3: Figure S2**. Interference of *LncRNA-TBP* promotes myoblast proliferation but inhibits myogenic differentiation. (**A**-**K**) Relative *LncRNA-TBP* expression (n = 6) (**A**), relative mRNA levels of several cell cycle genes (n = 6) (**B**), EdU proliferation assays (n = 3) (**C**), proliferation rate of myoblasts (n = 8) (**D**), CCK-8 assays (n = 6) (**E**), cell cycle analysis (n = 4) (**F**), MyHC immunostaining (n = 3) (**G**), myotube area (n = 8) (**H**), myoblast fusion index (n = 8) (**I**), relative mRNA (n = 6) (**J**) and protein (n = 3) (**K**) expression levels of myoblast differentiation marker genes with *LncRNA-TBP* interference in vitro. In panel (**K**), the numbers shown below the bands were folds of band intensities relative to control. Band intensities were quantified by ImageJ and normalized to GAPDH. Data are expressed as a fold-change relative to the control. Results are presented as mean ± SEM. In panels (**A**-**B**, **D**-**F**, and **H-J**), the statistical significance of differences between means was assessed using an independent sample t-test.

**Additional file 4: Figure S3**. Interference of *LncRNA-TBP* inhibits fatty acid oxidation in skeletal muscle. (**A-F**) Relative *LncRNA-TBP* expression (n = 4) (**A**), relative mtDNA content (n = 4) (**B**), relative fatty acid β-oxidation rate (n = 4) (**C**), relative FFA and TG content (n = 4) (**D**), relative mRNA (n = 6) (**E**) and protein (n = 3) (**F**) expression levels of fatty acid oxidation or synthesis related-genes in gastrocnemius with *LncRNA-TBP* interference in vivo. In panel (**F**), the numbers shown below the bands were folds of band intensities relative to control. Band intensities were quantified by ImageJ and normalized to GAPDH. Data are expressed as a fold-change relative to the control. Results are shown as mean ± SEM. In panels (**A**-**E**), the statistical significance of differences between means was assessed using an independent sample *t*-test.

**Additional file 5: Figure S4**. Interference of *LncRNA-TBP* activates fast-twitch muscle phenotype and reduces muscle hypertrophy. (**A**-**N**) relative enzymes activity of LDH and SDH (n = 4) (**A**), Relative glycogen content (n = 5) (**B**), relative mRNA expression levels of glycogenolytic and glycolytic genes (n = 6) (**C**), relative mRNA expression levels of several fast-/slow-twitch myofiber genes (n = 6) (**D**), immunohistochemistry analysis of MYH1A/MYH7B (n = 3) (**E**), MYH1A/MYH7B protein content (n = 8) (**F**), relative gastrocnemius muscle weight (n = 6) (**G**), H&E staining (n = 3) (**H**), relative mRNA (n = 6) (**I**), and the protein (n = 3) (**J**) expression levels of the atrophy and autophagy-related genes of in gastrocnemius with *LncRNA-TBP* interference in vivo. In panel (**J**), the numbers shown below the bands were folds of band intensities relative to control. Band intensities were quantified by ImageJ and normalized to GAPDH. Data are expressed as a fold-change relative to the control. Results are shown as mean ± SEM. In panels (**A**-**D**, **F** and **H-I**), the statistical significance of differences between means was assessed using an independent sample *t*-test.

**Additional file 6: Figure S5**. TBP specific target genes identified by ATAC-seq. (**A**) The RPISeq results showed that the TBP was predicted to interact with *LncRNA-TBP*. (**B**) Analysis of TBP-targeted binding target genes by ATAC-seq. (**C**) GO functions analysis of TBP specific binding target genes identified by ATAC-seq. (**D**) KEGG pathways analysis of TBP specific binding target genes identified by ATAC-seq.

**Additional file 7: Figure S6**. Overexpression and knockdown of *LncRNA-TBP* did not change the mRNA and protein expression level of *TBP*. (**A** and **B**) The mRNA level of *TBP* with *LncRNA-TBP* overexpression (n = 6) (**A**) and knockdown (n = 6) (**B**) in vitro. (**C** and **D**) The protein level of TBP with *LncRNA-TBP* overexpression (n = 3) (**C**) and knockdown (n = 3) (**D**) in vitro. In panel (**C, D**), the numbers shown below the bands were folds of band intensities relative to control. Band intensities were quantified by ImageJ and normalized to GAPDH. In panels (**A, B**), the statistical significance of differences between means was assessed using an independent sample *t*-test.

**Additional file 8: Figure S7**. Interference of *LncRNA-TBP* inhibit the transcriptional activity of TBP-target genes. (**A**-**G**) TBP enrichment at the *KLF4*, *GPI*, *TNNI2*, and *CDLN1A* promoter enrichment (n = 3) (**A**), relative promoter activity of *KLF4* (**B**), *GPI* (**C**), *TNNI2* (**D**), and *CDKN1A* (**E**) (n = 4), relative mRNA (n = 4) (**F**) and protein (n = 3) (**G**) of *KLF4*, *GPI*, *TNNI2*, and *CDKN1A* with *LncRNA-TBP* interference in vitro. In panel (**G**), the numbers shown below the bands were folds of band intensities relative to control. Band intensities were quantified by ImageJ and normalized to GAPDH. Data are expressed as a fold-change relative to the control. Results are shown as mean ± SEM. In panels (**A-F**), the statistical significance of differences between means was assessed using an independent sample *t*-test.

**Additional file 9: Figure S8**. The expression and location analysis of *TBP*. (**A**) Relative *TBP* expression during the proliferation and differentiation of CPM isolated from XH chicken (n = 4). (**B**) Subcellular location of TBP protein annotated by UniProt Knowledgebase (https://www.uniprot.org/). In panels (**A**), results are presented as mean ± SEM.

**Additional file 10.** Supplementary Information.

**Additional file 11: Table S2.** Comparative metabolome analysis of control group versus *LncRNA-TBP* overexpression gastrocnemius.

**Additional file 12: Table S3.** TBP specific target genes identified by ATAC-seq analysis.

## Data Availability

The data that support the findings of this study are available from the corresponding author upon reasonable request.

## Abbreviations

CPMs: Chicken primary myoblasts
CSA: Cross-section area
FAO: Fatty acid oxidation
FFA: Free fatty acid
HCA: Hierarchical clustering analysis
HE: Hematoxylin and eosin
LDH: Lactic dehydrogenase
mtDNA: Mitochondrial DNA
PEM: Pectoralis major
SDH: Succinate dehydrogenase
SOL: Soleus
TCA cycle: Tricarboxylic acid cycle
TG: Triglyceride
