# Supplementary material for: LncRNA-TBP mediates TATA-binding protein recruitment to regulate myogenesis and induce slow-twitch myofibers
Source: Cell Commun Signal. 2023 Jan 12;21:7. doi: 10.1186/s12964-022-01001-3 (PMC9835232; doi:10.1186/s12964-022-01001-3)

Figure 1H. LncRNA-TBP-ORF to Flag

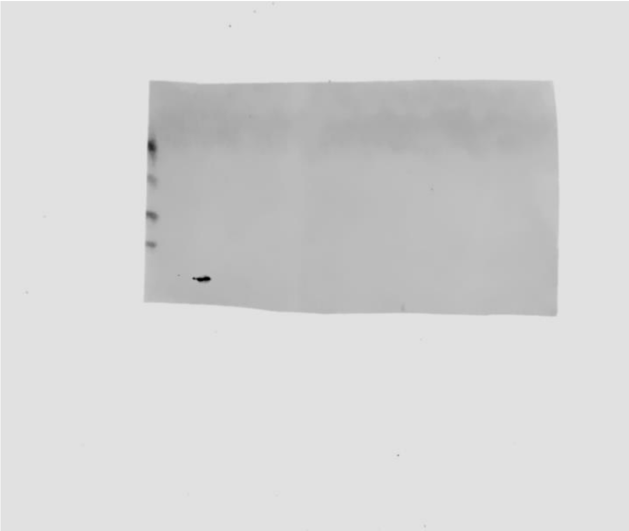

Figure 1H. LncRNA-TBP-ORF to Tubulin

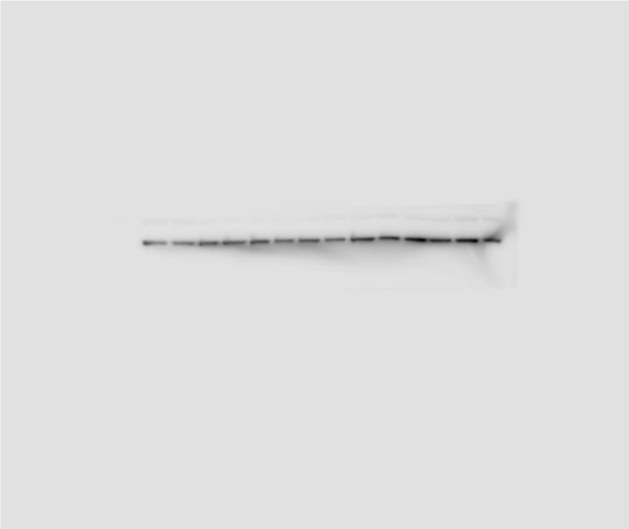

Figure 2H and S2H. ASO-LncRNA-TBP to MYHC (left) and pcDNA3.1-LncRNA-TBP to MYHC (right)

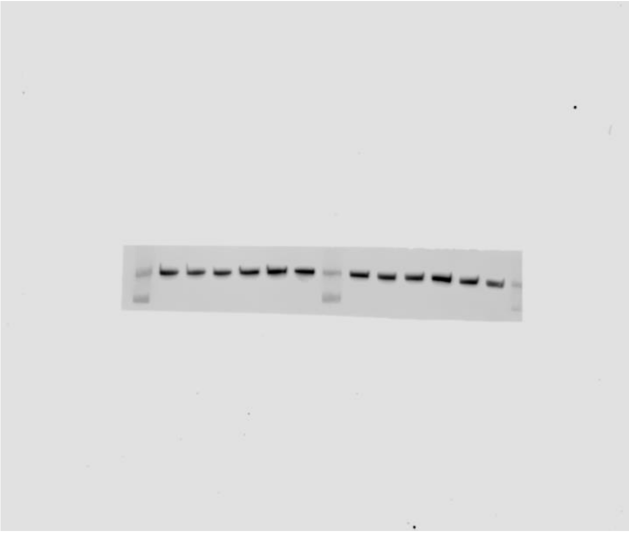

Figure 2H and S2H. ASO-LncRNA-TBP to Tubulin (right) and pcDNA3.1-LncRNA-TBP to Tubulin (left)

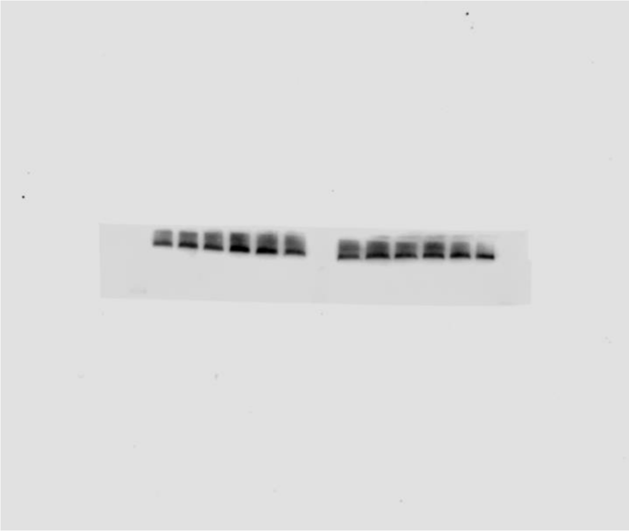

Figure 2H. pcDNA3.1-LncRNA-TBP to MYOD (right)

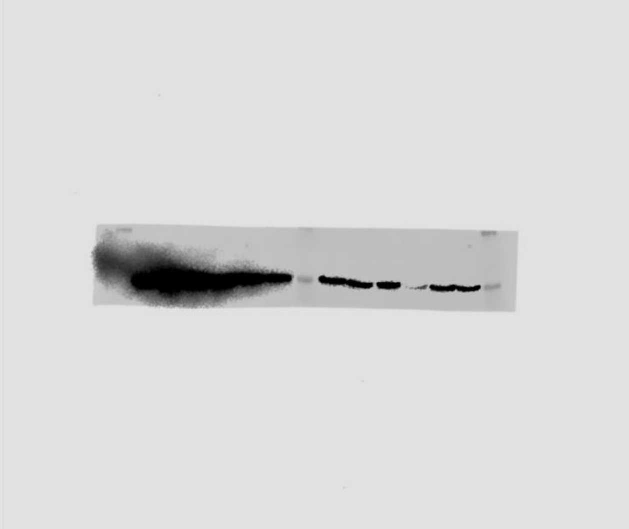

Figure 3F and S3F. Chol-ASO-LncRNA-TBP to CPT1 (left) and Lv-LncRNA-TBP to CPT1 (right)

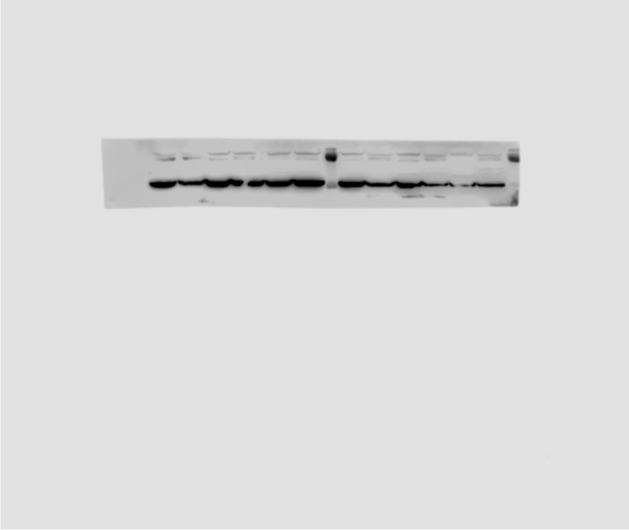

Figure 3F and S3F. Chol-ASO-LncRNA-TBP to FASN (left) and Lv-LncRNA-TBP to FASN (right)

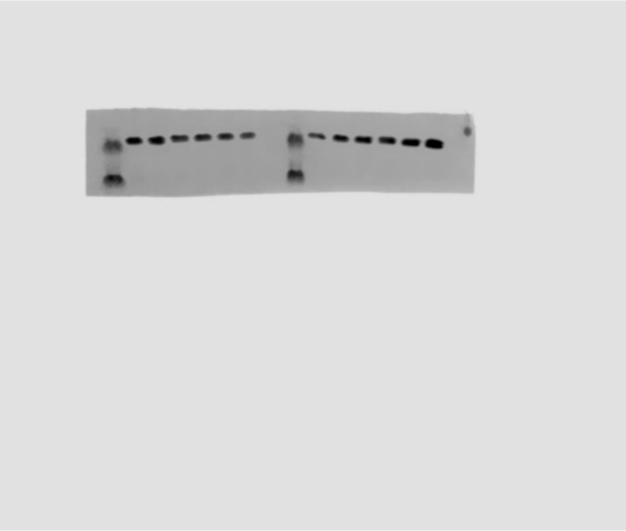

Figure 3F. Lv-LncRNA-TBP to GAPDH (left)

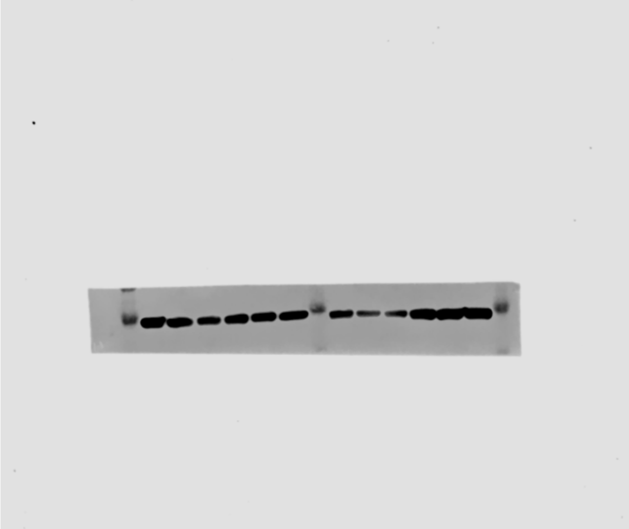

Figure 4J and S4J. Chol-ASO-LncRNA-TBP to P62 (right) and Lv-LncRNA-TBP to P62 (left)

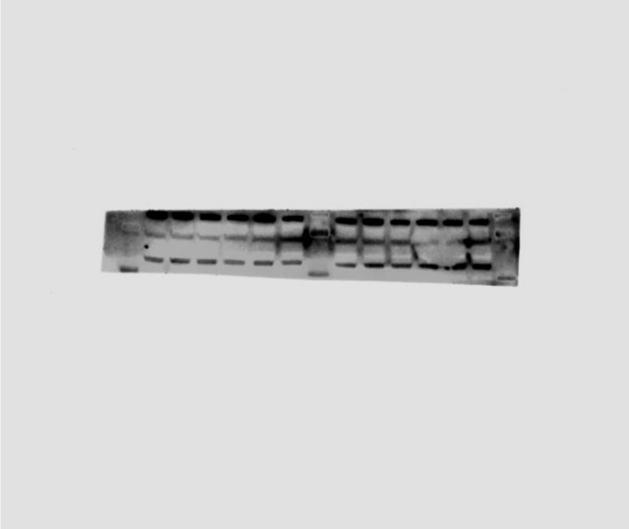

Figure 4J. Lv-LncRNA-TBP to GAPDH (right)

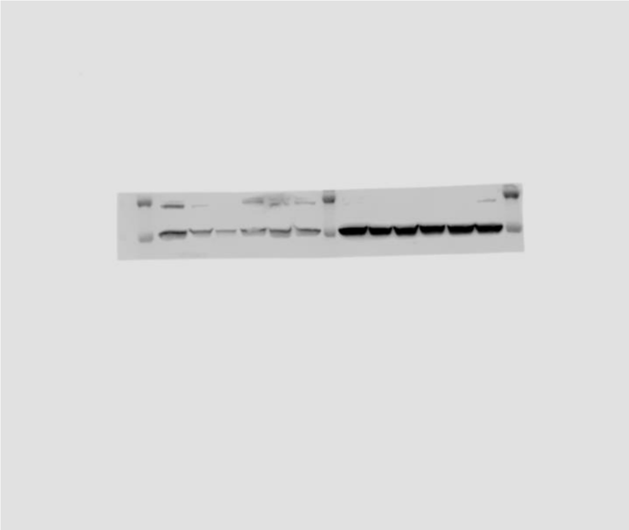

Figure 4J. Lv-LncRNA-TBP to LC3B (right)

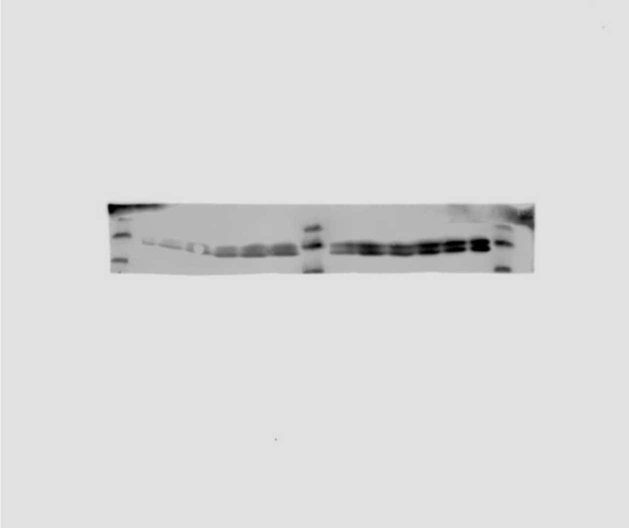

Figure 4J. Lv-LncRNA-TBP to ULK1 (right)

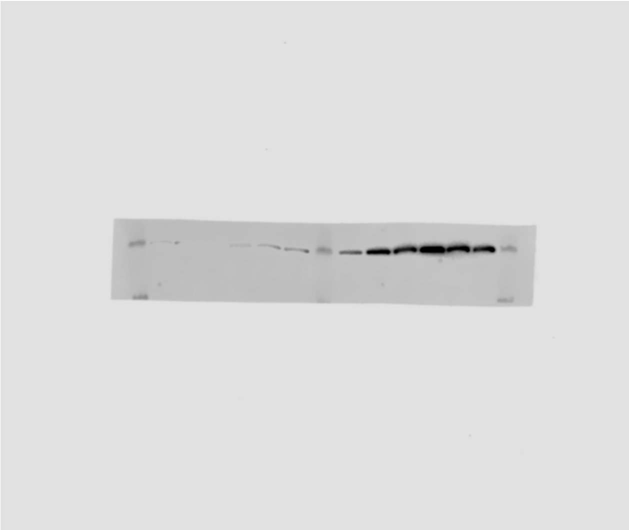

Figure 5K and S7G. ASO-LncRNA-TBP to CDKN1A (left) and pcDNA3.1-LncRNA-TBP to CDKN1A (right)

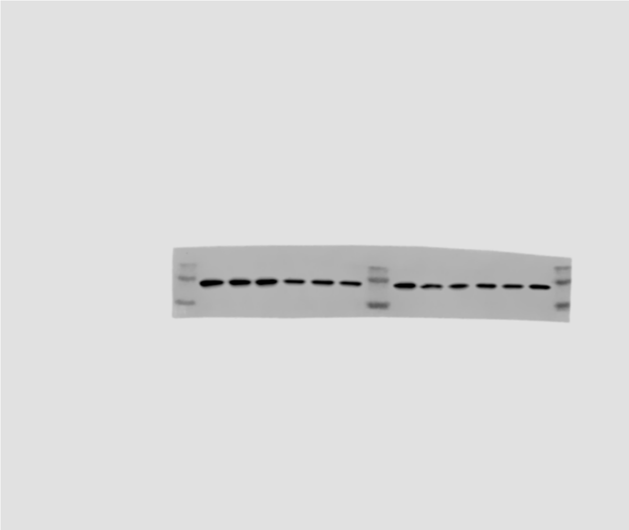

Figure 5K and S7G. ASO-LncRNA-TBP to KLF4 (left) and pcDNA3.1-LncRNA-TBP to KLF4 (right)

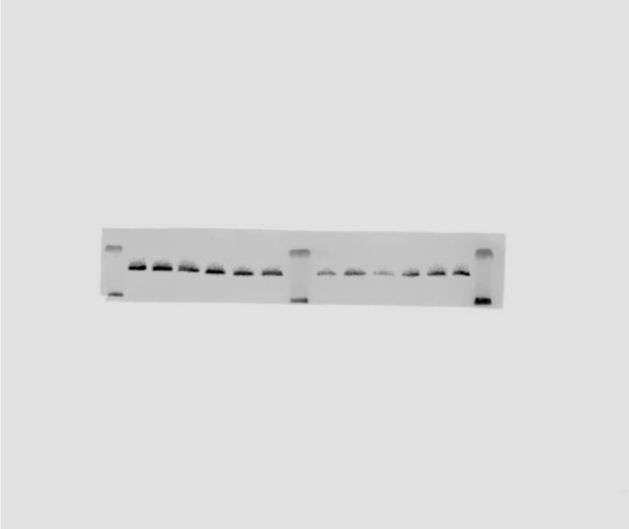

Figure 5K. pcDNA3.1-LncRNA-TBP to GAPDH (left)

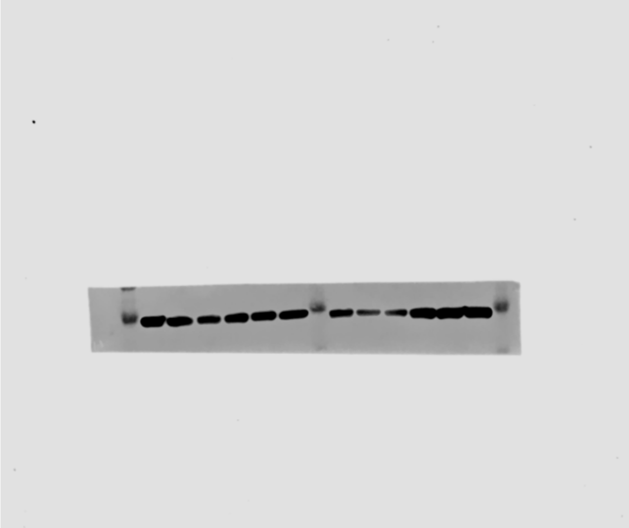

Figure 5K. pcDNA3.1-LncRNA-TBP to GPI (left)

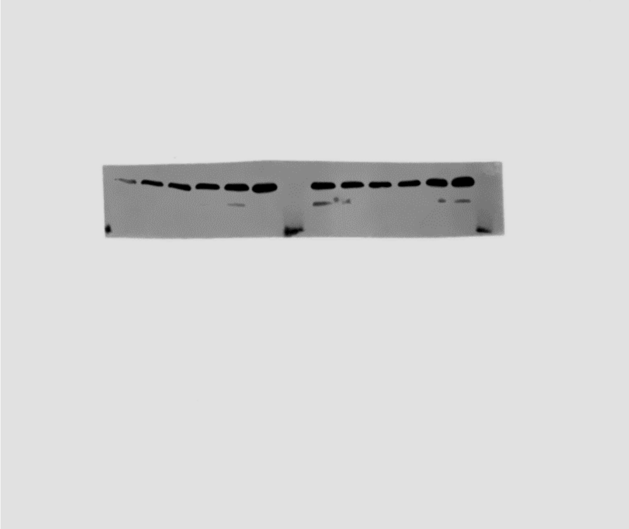

Figure 5K. pcDNA3.1-LncRNA-TBP to TNNI2 (right)

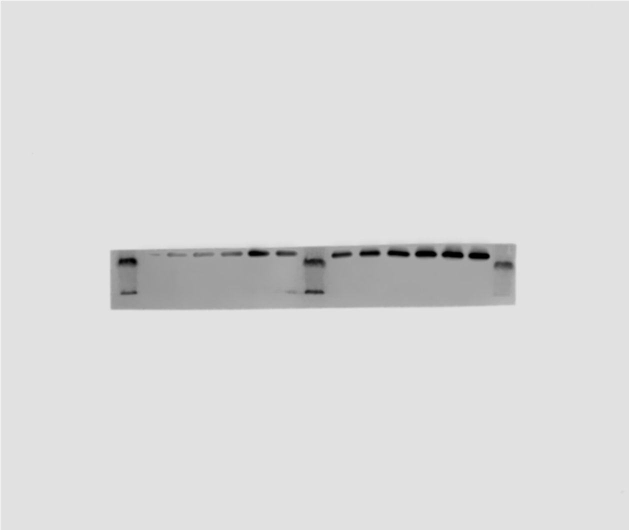

Figure 6J. pcDNA3.1-TBP to MYOD

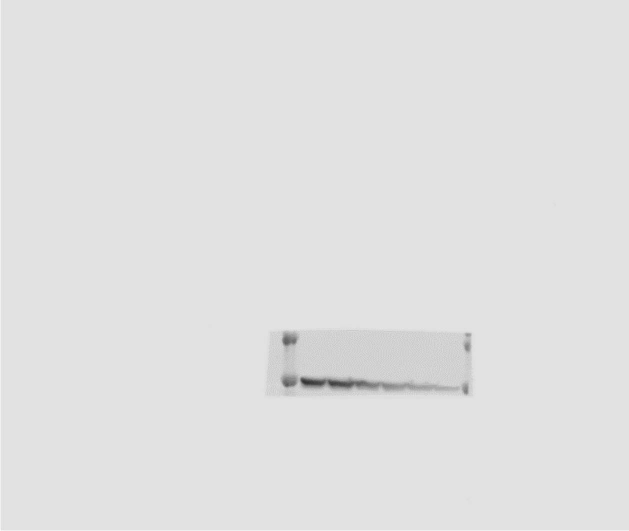

Figure 6J and 6T. si-TBP to GAPDH (right) and pcDNA3.1-TBP to GAPDH (left)

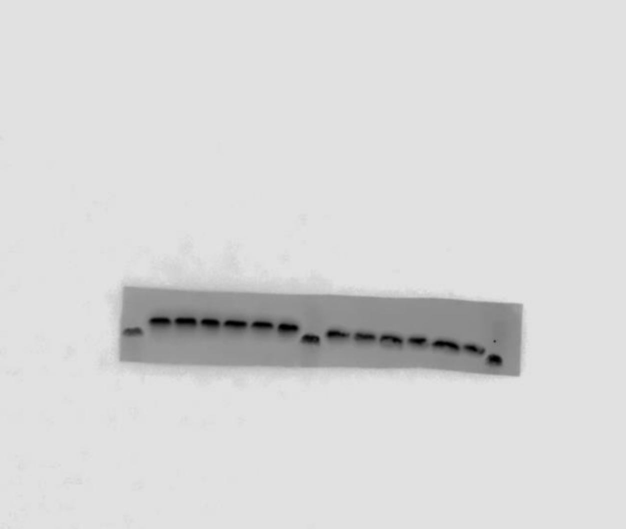

Figure 6J and 6T. si-TBP to MYHC (right) and pcDNA3.1-TBP to MYHC (left)

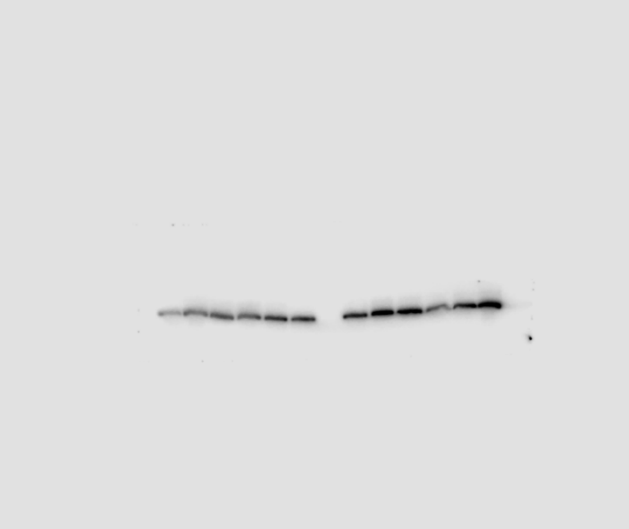

Figure 6T. si-TBP to MYOD

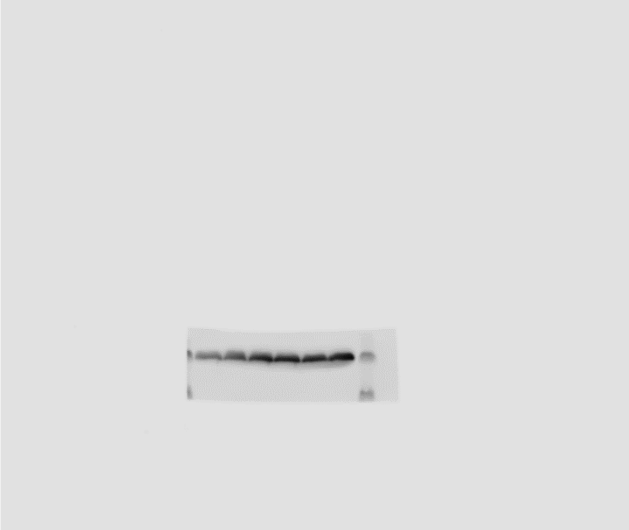

Figure S2H. ASO-LncRNA-TBP to MYOD (left)

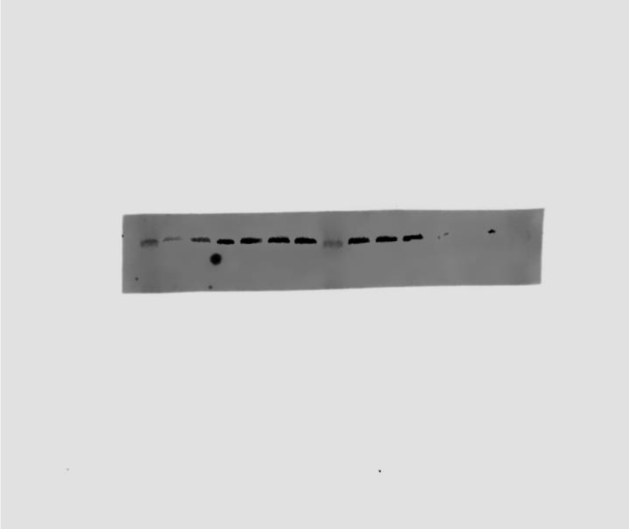

Figure S3F. Chol-ASO-LncRNA-TBP to GAPDH (left)

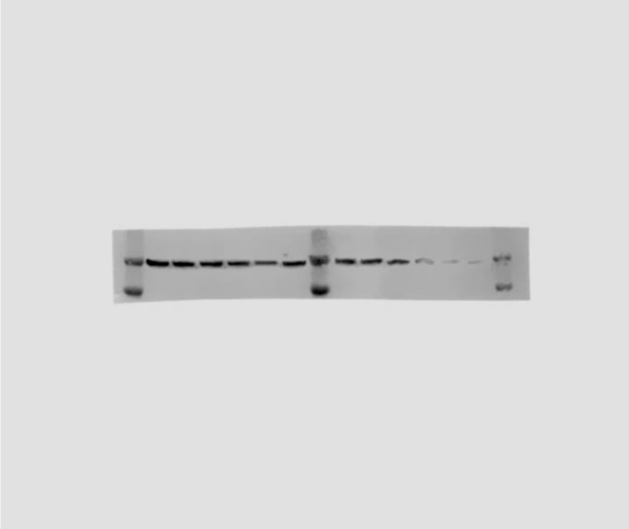

Figure S4J. Chol-ASO-LncRNA-TBP to GAPDH (left)

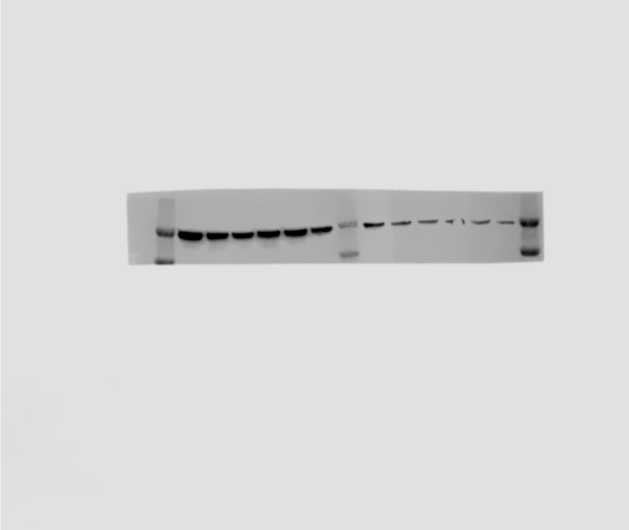

Figure S4J. Chol-ASO-LncRNA-TBP to LC3B (left)

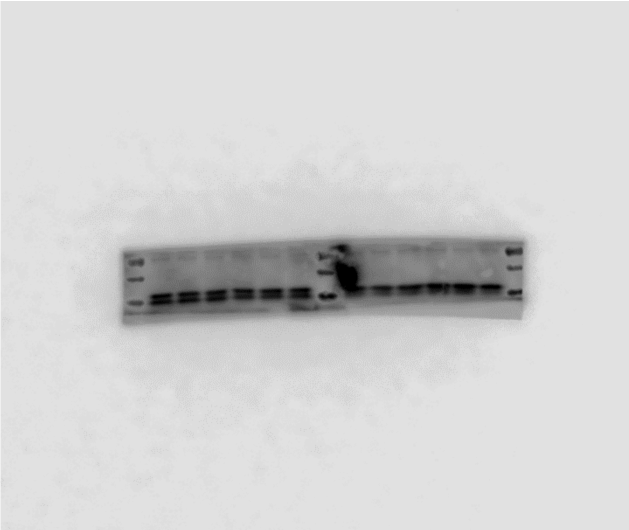

Figure S4J. Chol-ASO-LncRNA-TBP to ULK1 (right)

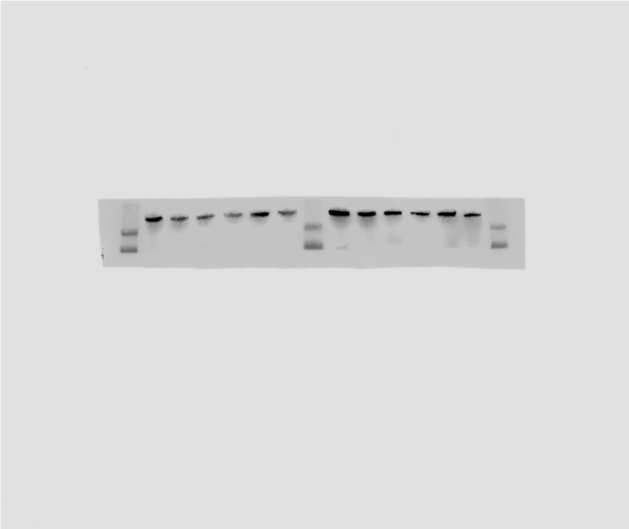

Figure S6C and S6D. ASO-LncRNA-TBP to TBP (right) and pcDNA3.1-LncRNA-TBP to TBP (left)

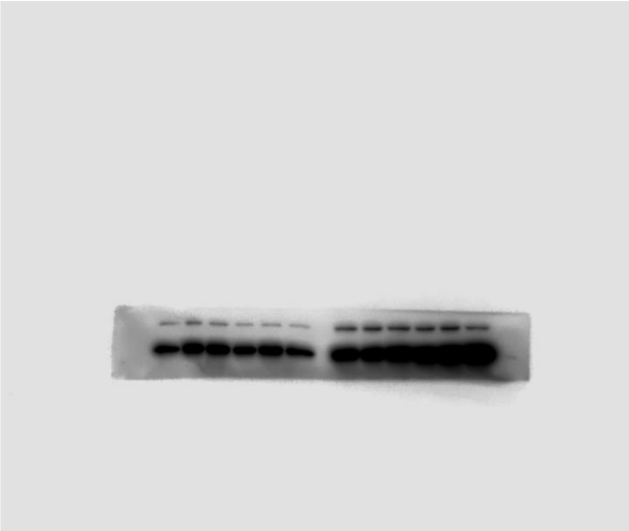

Figure S6C and S6D. ASO-LncRNA-TBP to Tubulin (right) and pcDNA3.1-LncRNA-TBP to Tubulin (left)

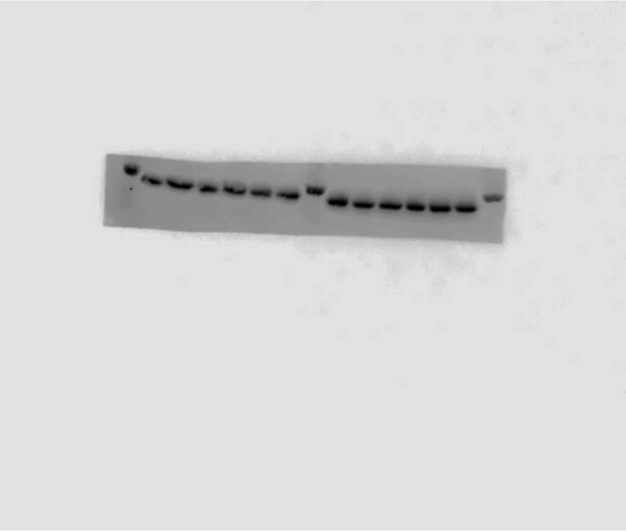

Figure S7G. ASO-LncRNA-TBP to GAPDH (right)

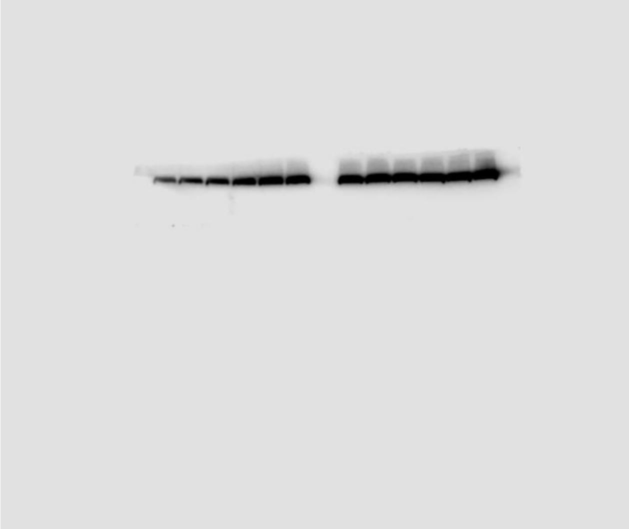

Figure S7G. ASO-LncRNA-TBP to GPI (left)

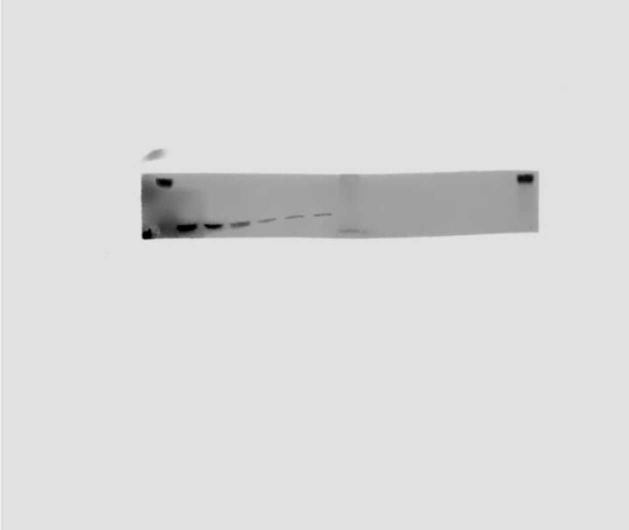

G. ASO-LncRNA-TBP to TNNI2 (left)

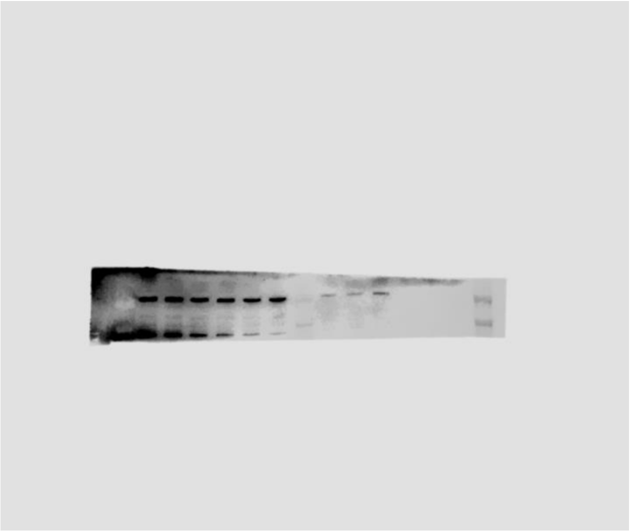

Supplement: Supplementary file 2 — Additional file 1. Original data of WB. [file 12964_2022_1001_MOESM2_ESM.pdf]
