# Supplementary figures and images for: LncRNA-TBP mediates TATA-binding protein recruitment to regulate myogenesis and induce slow-twitch myofibers
Source: Cell Commun Signal. 2023 Jan 12;21:7. doi: 10.1186/s12964-022-01001-3 (PMC9835232; doi:10.1186/s12964-022-01001-3)

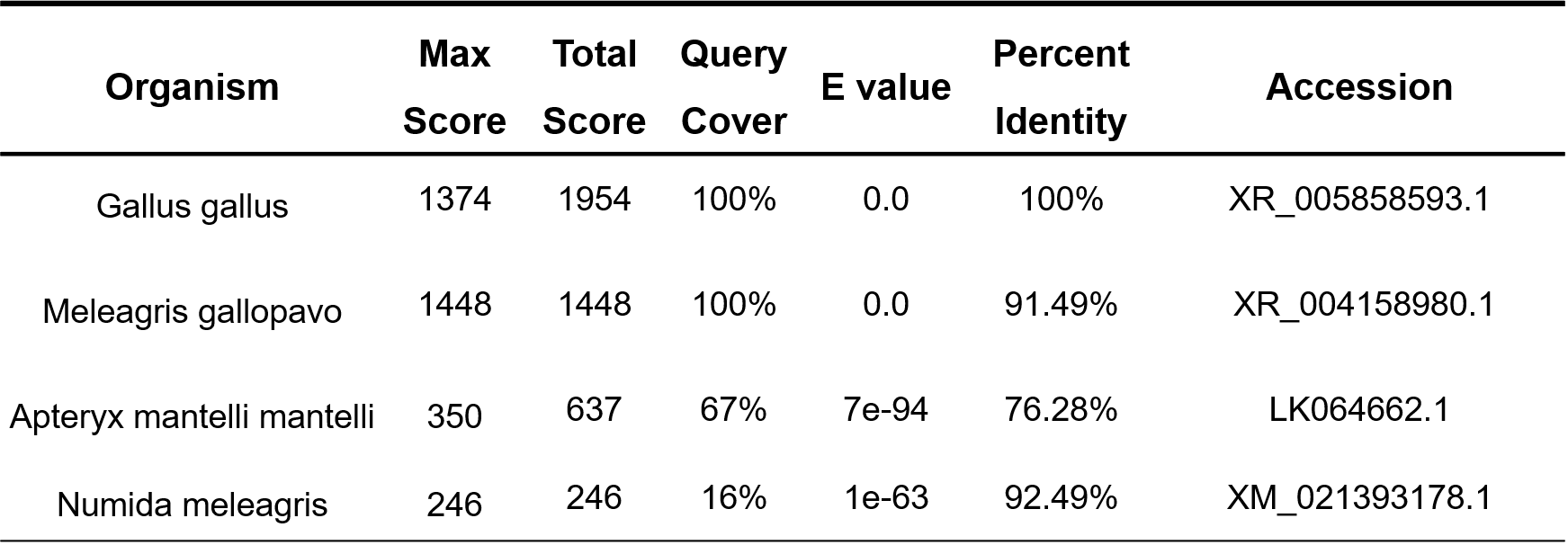

Supplement: Supplementary file 3 — Additional file 2: Figure S1. Conservative analysis of LncRNA-TBP performed by using the NCBI’s BLAST. A total of eighteen species, including Anas platyrhynchos, Anser cygnoides, Apteryx mantelli mantelli, Aquila chrysaetos, Bos taurus, Coturnix japonica, Gallus gallus, Geospiza fortis, Homo sapiens, Meleagris gallopavo, Melopsittacus undulatus, Mus musculus, Numida meleagris, Ovis aries, Pan troglodytes, Rattus norvegicus, Sus scrofa and Zebra finch were used for Nucleotide BLAST. Top 4 most conservative results were listed above. [file 12964_2022_1001_MOESM3_ESM.tif]

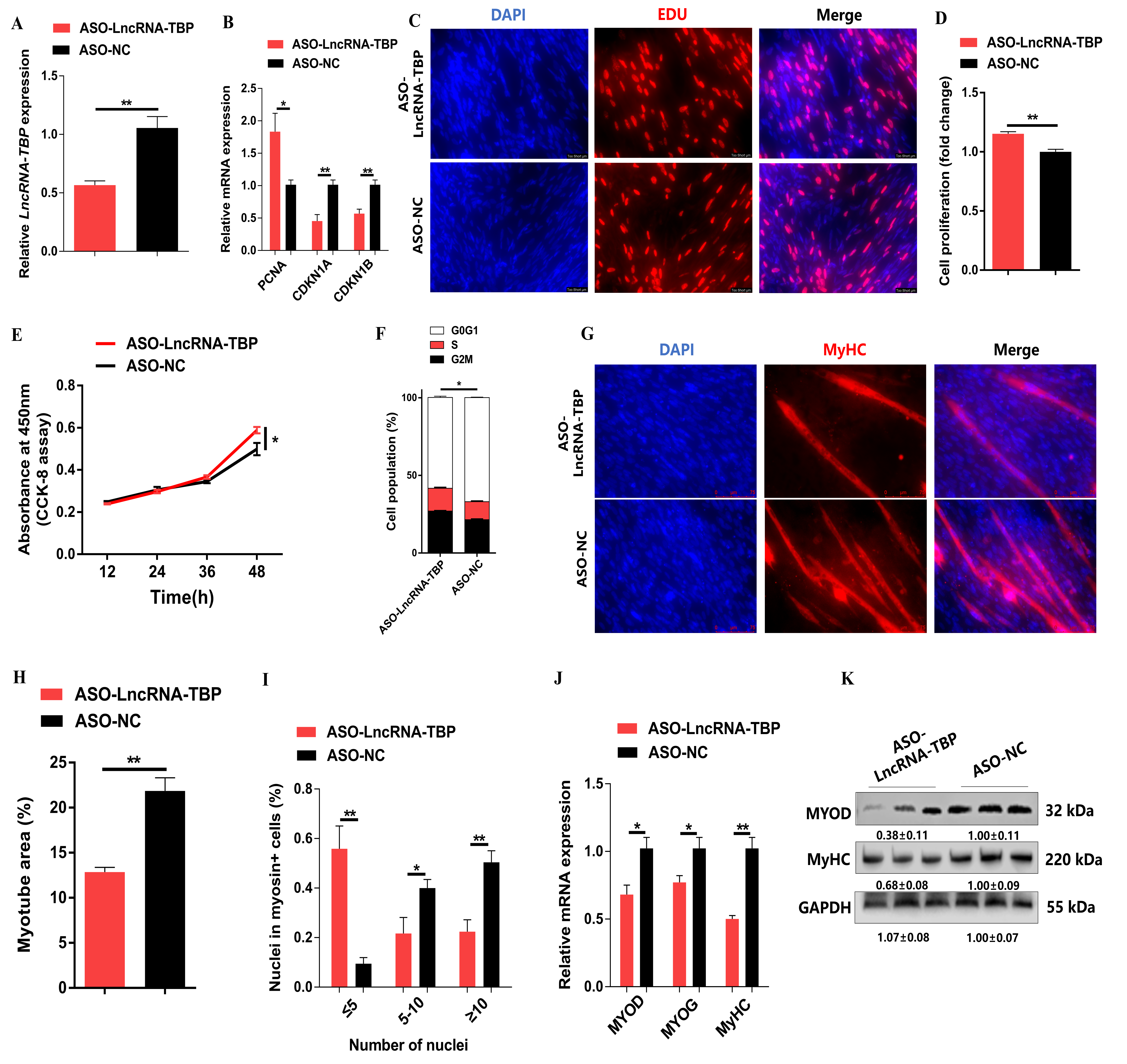

Supplement: Supplementary file 4 — Additional file 3: Figure S2. Interference of LncRNA-TBP promotes myoblast proliferation but inhibits myogenic differentiation. (A-K) Relative LncRNA-TBP expression (n = 6) (A), relative mRNA levels of several cell cycle genes (n = 6) (B), EdU proliferation assays (n = 3) (C), proliferation rate of myoblasts (n = 8) (D), CCK-8 assays (n = 6) (E), cell cycle analysis (n = 4) (F), MyHC immunostaining (n = 3) (G), myotube area (n = 8) (H), myoblast fusion index (n = 8) (I), relative mRNA (n = 6) (J) and protein (n = 3) (K) expression levels of myoblast differentiation marker genes with LncRNA-TBP interference in vitro. In panel (K), the numbers shown below the bands were folds of band intensities relative to control. Band intensities were quantified by ImageJ and normalized to GAPDH. Data are expressed as a fold-change relative to the control. Results are presented as mean ± SEM. In panels (A-B, D-F, and H-J), the statistical significance of differences between means was assessed using an independent sample t-test. [file 12964_2022_1001_MOESM4_ESM.tif]

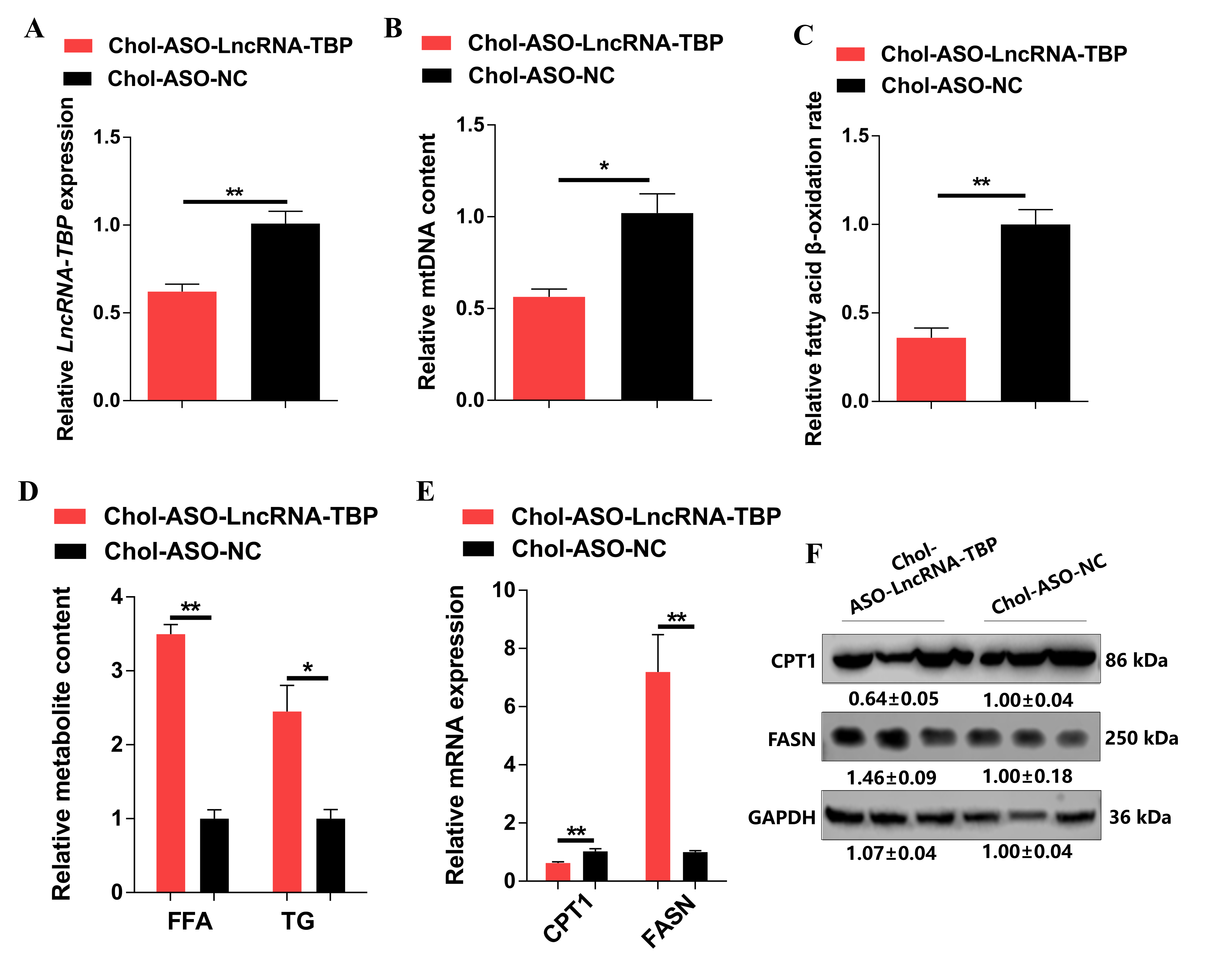

Supplement: Supplementary file 5 — Additional file 4: Figure S3. Interference of LncRNA-TBP inhibits fatty acid oxidation in skeletal muscle. (A-F) Relative LncRNA-TBP expression (n = 4) (A), relative mtDNA content (n = 4) (B), relative fatty acid β-oxidation rate (n = 4) (C), relative FFA and TG content (n = 4) (D), relative mRNA (n = 6) (E) and protein (n = 3) (F) expression levels of fatty acid oxidation or synthesis related-genes in gastrocnemius with LncRNA-TBP interference in vivo. In panel (F), the numbers shown below the bands were folds of band intensities relative to control. Band intensities were quantified by ImageJ and normalized to GAPDH. Data are expressed as a fold-change relative to the control. Results are shown as mean ± SEM. In panels (A-E), the statistical significance of differences between means was assessed using an independent sample t-test. [file 12964_2022_1001_MOESM5_ESM.tif]

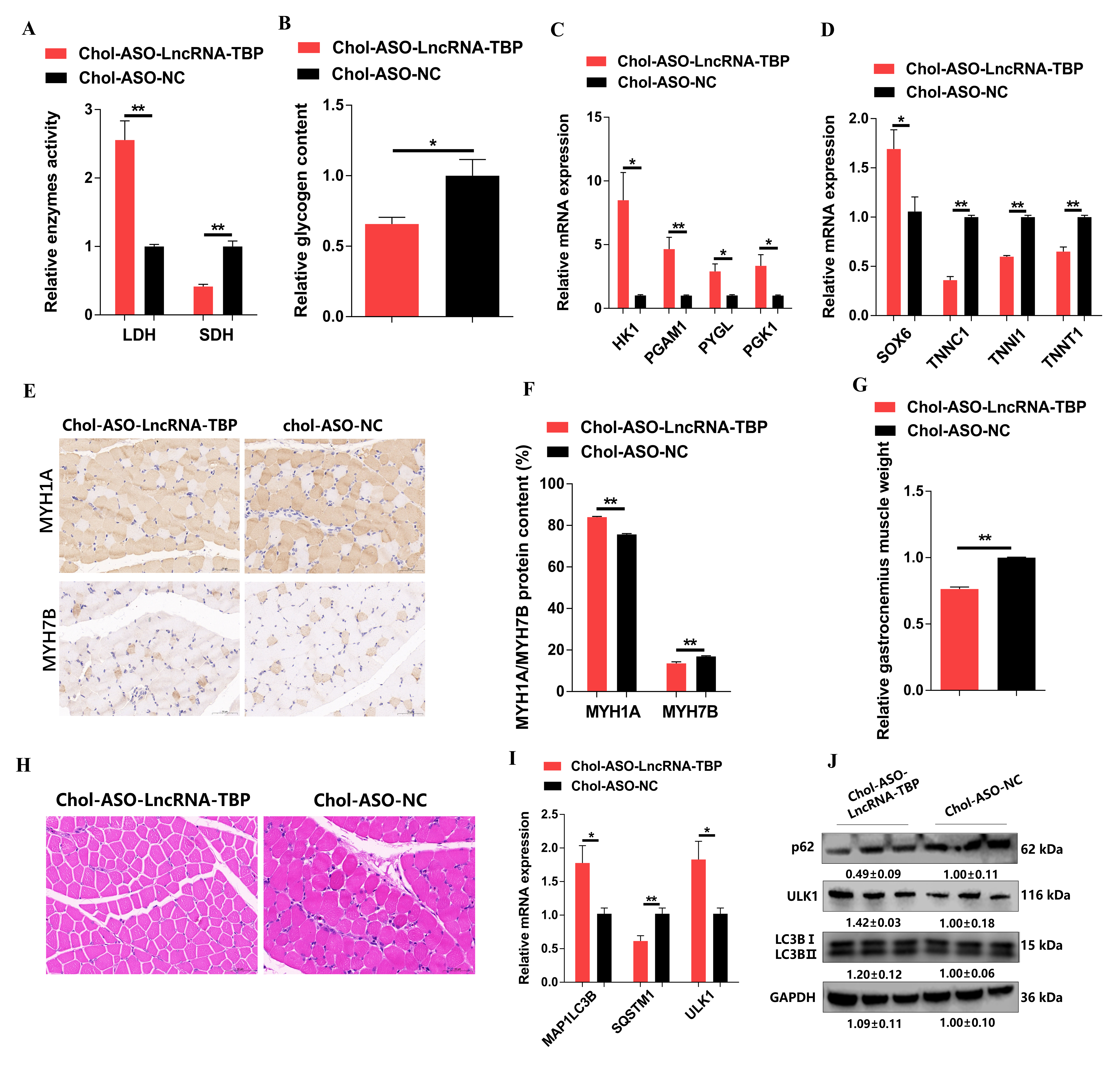

Supplement: Supplementary file 6 — Additional file 5: Figure S4. Interference of LncRNA-TBP activates fast-twitch muscle phenotype and reduces muscle hypertrophy. (A-N) relative enzymes activity of LDH and SDH (n = 4) (A), Relative glycogen content (n = 5) (B), relative mRNA expression levels of glycogenolytic and glycolytic genes (n = 6) (C), relative mRNA expression levels of several fast-/slow-twitch myofiber genes (n = 6) (D), immunohistochemistry analysis of MYH1A/MYH7B (n = 3) (E), MYH1A/MYH7B protein content (n = 8) (F), relative gastrocnemius muscle weight (n = 6) (G), H&E staining (n = 3) (H), relative mRNA (n = 6) (I), and the protein (n = 3) (J) expression levels of the atrophy and autophagy-related genes of in gastrocnemius with LncRNA-TBP interference in vivo. In panel (J), the numbers shown below the bands were folds of band intensities relative to control. Band intensities were quantified by ImageJ and normalized to GAPDH. Data are expressed as a fold-change relative to the control. Results are shown as mean ± SEM. In panels (A-D, F and H-I), the statistical significance of differences between means was assessed using an independent sample t-test. [file 12964_2022_1001_MOESM6_ESM.tif]

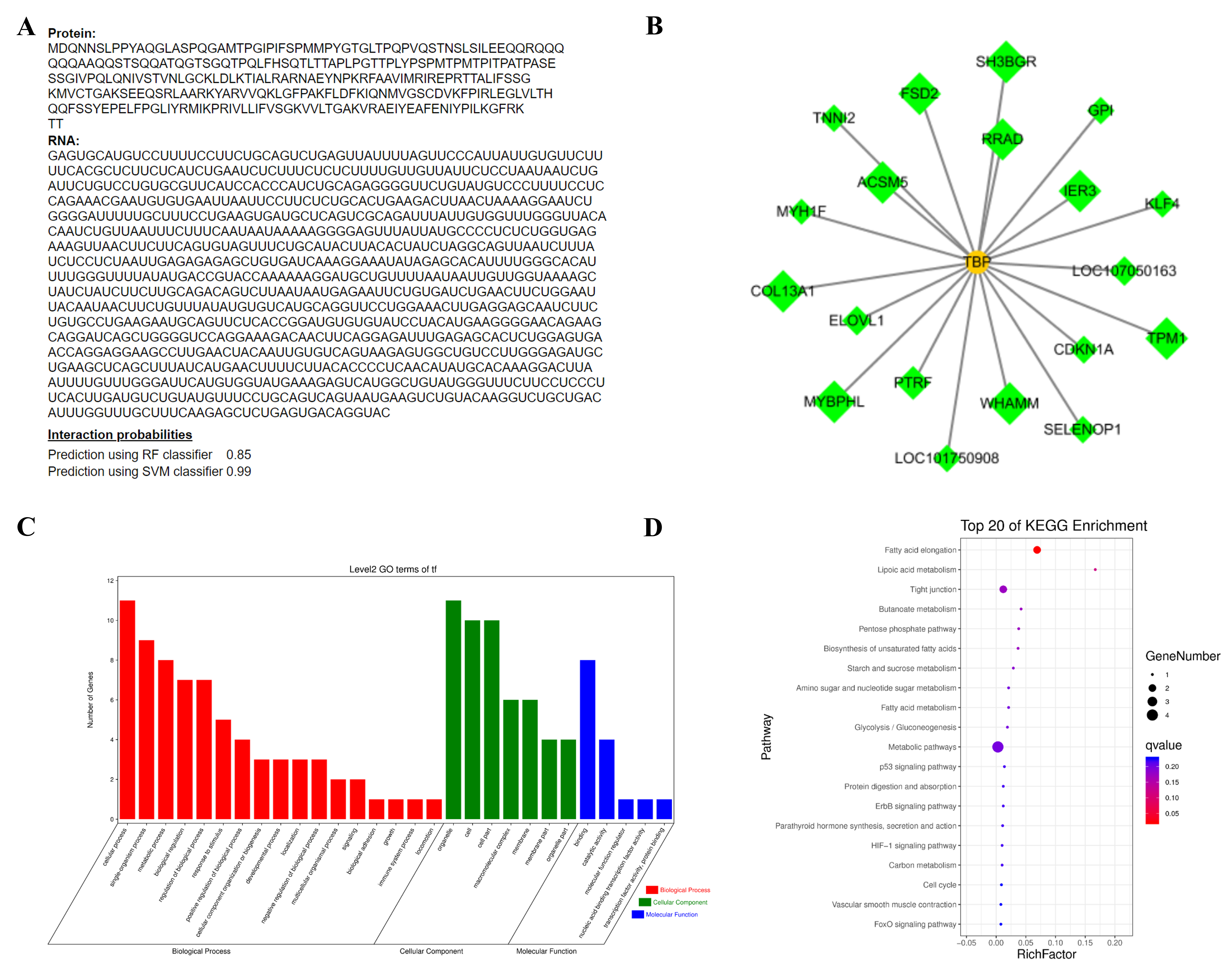

Supplement: Supplementary file 7 — Additional file 6: Figure S5. TBP specific target genes identified by ATAC-seq. (A) The RPISeq results showed that the TBP was predicted to interact with LncRNA-TBP. (B) Analysis of TBP-targeted binding target genes by ATAC-seq. (C) GO functions analysis of TBP specific binding target genes identified by ATAC-seq. (D) KEGG pathways analysis of TBP specific binding target genes identified by ATAC-seq. [file 12964_2022_1001_MOESM7_ESM.tif]

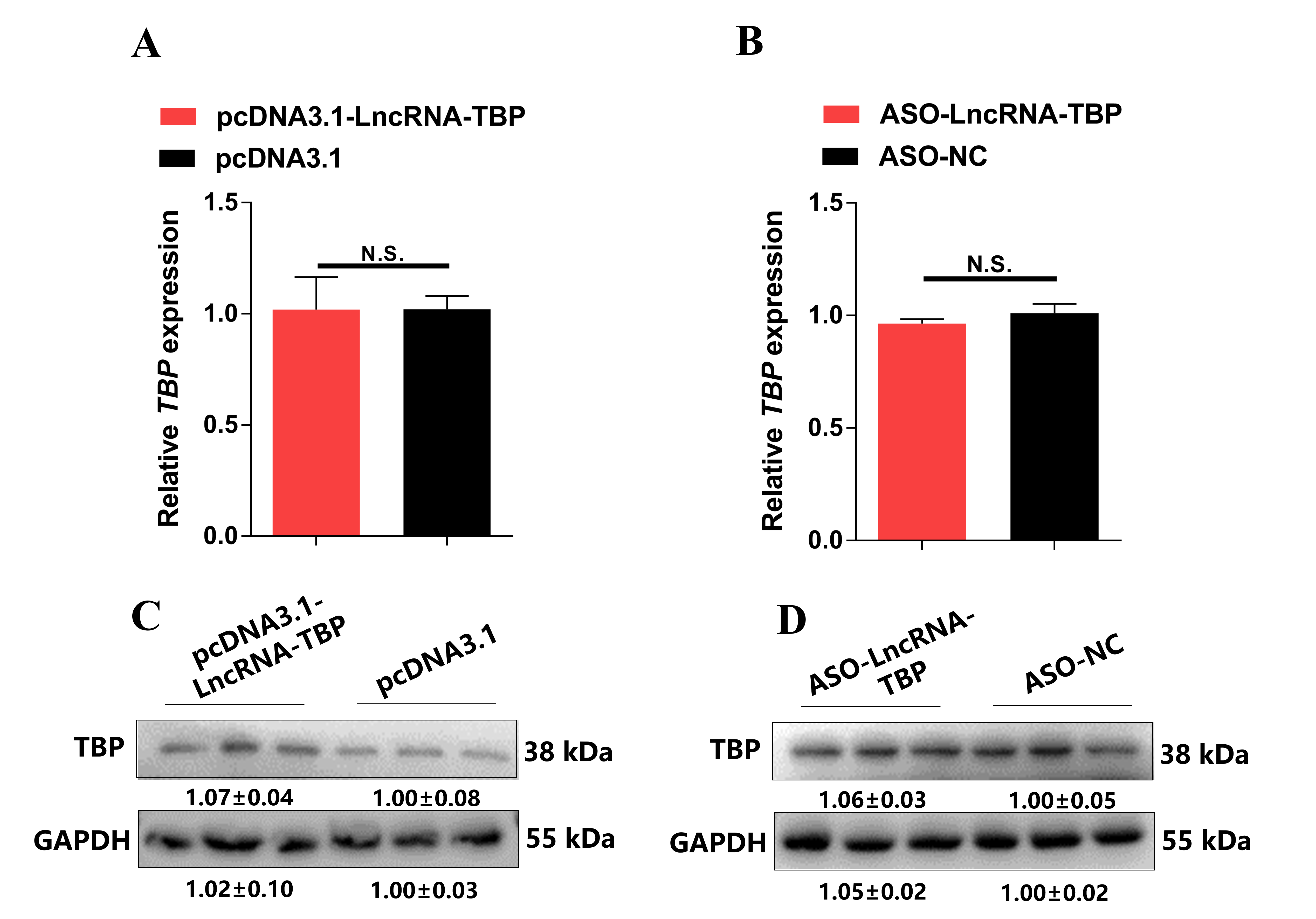

Supplement: Supplementary file 8 — Additional file 7: Figure S6. Overexpression and knockdown of LncRNA-TBP did not change the mRNA and protein expression level of TBP. (A and B) The mRNA level of TBP with LncRNA-TBP overexpression (n = 6) (A) and knockdown (n = 6) (B) in vitro. (C and D) The protein level of TBP with LncRNA-TBP overexpression (n = 3) (C) and knockdown (n = 3) (D) in vitro. In panel (C, D), the numbers shown below the bands were folds of band intensities relative to control. Band intensities were quantified by ImageJ and normalized to GAPDH. In panels (A, B), the statistical significance of differences between means was assessed using an independent sample t-test. [file 12964_2022_1001_MOESM8_ESM.tif]

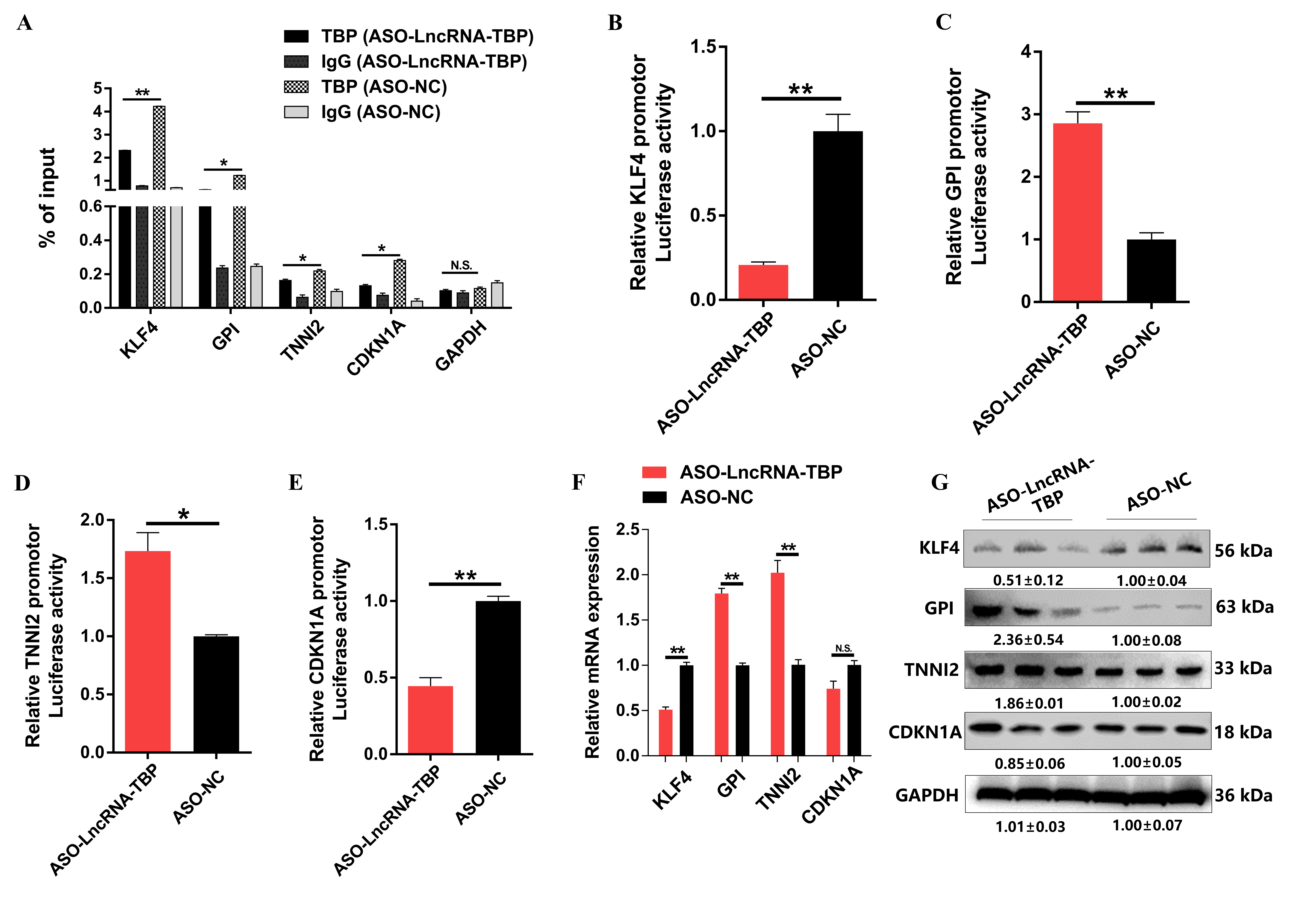

Supplement: Supplementary file 9 — Additional file 8: Figure S7. Interference of LncRNA-TBP inhibit the transcriptional activity of TBP-target genes. (A-G) TBP enrichment at the KLF4, GPI, TNNI2, and CDLN1A promoter enrichment (n = 3) (A), relative promoter activity of KLF4 (B), GPI (C), TNNI2 (D), and CDKN1A (E) (n = 4), relative mRNA (n = 4) (F) and protein (n = 3) (G) of KLF4, GPI, TNNI2, and CDKN1A with LncRNA-TBP interference in vitro. In panel (G), the numbers shown below the bands were folds of band intensities relative to control. Band intensities were quantified by ImageJ and normalized to GAPDH. Data are expressed as a fold-change relative to the control. Results are shown as mean ± SEM. In panels (A-F), the statistical significance of differences between means was assessed using an independent sample t-test. [file 12964_2022_1001_MOESM9_ESM.tif]

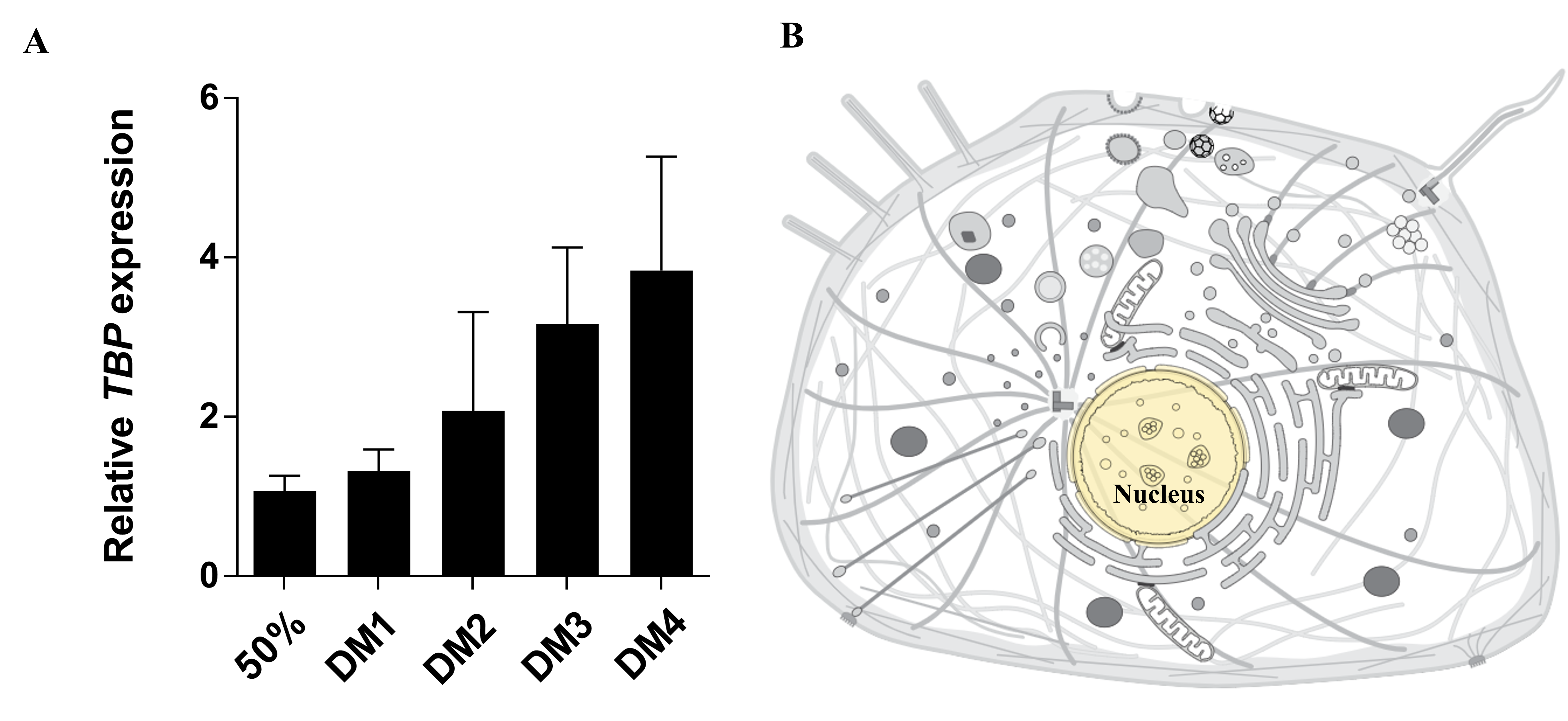

Supplement: Supplementary file 10 — Additional file 9: Figure S8. The expression and location analysis of TBP. (A) Relative TBP expression during the proliferation and differentiation of CPM isolated from XH chicken (n = 4). (B) Subcellular location of TBP protein annotated by UniProt Knowledgebase (https://www.uniprot.org/). In panels (A), results are presented as mean ± SEM. [file 12964_2022_1001_MOESM10_ESM.tif]
