# Supplementary material for: LncRNA-TBP mediates TATA-binding protein recruitment to regulate myogenesis and induce slow-twitch myofibers
Source: Cell Commun Signal. 2023 Jan 12;21:7. doi: 10.1186/s12964-022-01001-3 (PMC9835232; doi:10.1186/s12964-022-01001-3)
Supplement: Supplementary file 11 — Additional file 10. Supplementary Information. [file 12964_2022_1001_MOESM11_ESM.docx]

**Supplementary Table 1.** The full-length sequence of *LncRNA-TBP*. Coordinates are listed according to GRCg6a reference, Annotation Release 104 (chromosome 3: 82341588 to 82342330, 82369736 to 82370049).

GAGTGCATGTCCTTTTCCTTCTGCAGTCTGAGTTATTTTAGTTCCCATTATTGTGTTCTTTTCACGCTCTTCTCATCTGAATCTCTTTCTCTCTTTTGTTGTTATTCTCCTAATAATCTGATTCTGTCCTGTGCGTTCATCCACCCATCTGCAGAGGGGTTCTGTATGTCCCTTTTCCTCCAGAAACGAATGTGTGAATTAATTCCTTCTCTGCACTGAAGACTTAACTAAAAGGAATCTGGGGATTTTTGCTTTCCTGAAGTGATGCTCAGTCGCAGATTTATTGTGGTTTGGGTTACACAATCTGTTAATTTCTTTCAATAATAAAAAGGGGAGTTTATTATGCCCCTCTCTGGTGAGAAAGTTAACTTCTTCAGTGTAGTTTCTGCATACTTACACTATCTAGGCAGTTAATCTTTATCTCCTCTAATTGAGAGAGAGCTGTGATCAAAGGAAATATAGAGCACATTTTGGGCACATTTTGGGTTTTATATGACCGTACCAAAAAAGGATGCTGTTTTAATAATTGTTGGTAAAAGCTATCTATCTTCTTGCAGACAGTCTTAATAATGAGAATTCTGTGATCTGAACTTCTGGAATTACAATAACTTCTGTTTATATGTGTCATGCAGGTTCCTGGAAACTTGAGGAGCAATCTTCTGTGCCTGAAGAATGCAGTTCTCACCGGATGTGTGTATCCTACATGAAGGGGAACAGAAGCAGGATCAGCTGGGGTCCAGGAAAGACAACTTCAGGAGATTTGAGAGCACTCTGGAGTGAACCAGGAGGAAGCCTTGAACTACAATTGTGTCAGTAAGAGTGGCTGTCCTTGGGAGATGCTGAAGCTCAGCTTTATCATGAACTTTTCTTACACCCCTCAACATATGCACAAAGGACTTAATTTTGTTTGGGATTCATGTGGTATGAAAGAGTCATGGCTGTATGGGTTTCTTCCTCCCTTCACTTGATGTCTGTATGTTTCCTGCAGTCAGTAATGAAGTCTGTACAAGGTCTGCTGACATTTGGTTTGCTTTCAAGAGCTCTGAGTGACAGGTAC

**Supplementary Table 4.** Information of Primers.

| **Primer name** | **Primer sequences (5’ to 3’)** | **Usage** |
| --- | --- | --- |
| qPCR-LncRNA-TBP | F: ACTTGAGGAGCAATCTTCTGTG | qPCR |
|  | R: ATCTCCCAAGGACAGCCAC |  |
| qPCR-TBP | F: ACGGTGAATCTTGGTTGC | qPCR |
|  | R: TCTTGCGTACTTCCTTGC |  |
| qPCR-PCNA | F: GTGCTGGGACCTGGGTT | qPCR |
|  | R: CGTATCCGCATTGTCTTCT |  |
| qPCR-CDKN1A | F: CCCGTAGACCACGAGCAGAT | qPCR |
|  | R: CGTCTCGGTCTCGAAGTTGA |  |
| qPCR-CDKN1B | F: TCGCTGTGCTGGGCTGAA | qPCR |
|  | R: CAAGGACGAAAGGATGTGGG |  |
| qPCR-MYOD | F: GCTACTACACGGAATCACCAAAT | qPCR |
|  | R: CTGGGCTCCACTGTCACTCA |  |
| qPCR-MYOG | F: CGGAGGCTGAAGAAGGTGAA | qPCR |
|  | R: CGGTCCTCTGCCTGGTCAT |  |
| qPCR-MyHC | F: CTCCTCACGCTTTGGTAA | qPCR |
|  | R: TGATAGTCGTATGGGTTGGT |  |
| qPCR-COX2 | F: GTAGATGCCCAAGAAGTT | qPCR |
|  | R: GTTTGATTTAGTCGTCCAG |  |
| qPCR-β-globin | F: CAGCCAGGTGGAGGATTT | qPCR |
|  | R: GAATAGGAGGACCCTCTGTTAG |  |
| qPCR-CPT1 | F: GCTTATTGTAGTTGTGGGTG | qPCR |
|  | R: AAAGTTTGCCGTGTTCAG |  |
| qPCR-FASN | F: CGCAGGCATAGCAGGAAA | qPCR |
|  | R: CCAAAGAAGGAGGCATCAA |  |
| qPCR-HK1 | F: CTGGATCTCGGTGGTTCTTAC | qPCR |
|  | R: TTGTCGGCACGGGAAAGA |  |
| qPCR-PGAM1 | F: GCGAGGCTCAGGTGAAGAT | qPCR |
|  | R: GTCCTCCGTCAGGTCAGC |  |
| qPCR-PGK1 | F: CCCTGGATAAGGTGGATG | qPCR |
|  | R: TTGTCAGGCATGGGAACT |  |
| qPCR-PYGL | F: ACATTTGCCTACACGAACC | qPCR |
|  | R: TGCCTCCCTCCTCTATCA |  |
| qPCR-SOX6 | F: TCAGGTTCAGGGTCACATGCC | qPCR |
|  | R: TTGCTGGAGCTGTAAAGGGC |  |
| qPCR-TNNC1 | F: GTTGAGCAGTTGACAGAAGA | qPCR |
|  | R: GAACCATCATAACAAGGAAC |  |
| qPCR-TNNI1 | F: GAGGAGTGGGAGCAGGAGAT | qPCR |
|  | R: TTCGTCCACAATCTCAACCT |  |
| qPCR-TNNT1 | F: GAGCCGCACGGAGAAGGAGC | qPCR |
|  | R: CCCGAAGTGGGGCATGTTGG |  |
| qPCR-ATROGIN1 | F: TCAACGGGTCGGCAAGTCT | qPCR |
|  | R: TCCCTCCCATCGCTCAGTC |  |
| qPCR-LC3B | F: GAGCAAAGAGTTGAAGATG | qPCR |
|  | R: GTCCTAGACGGAAGATTG |  |
| qPCR-SQSTM1 | F: AGCGACGAGGAGCTGGATC | qPCR |
|  | R: CCTTGTGGATGCCTTTACCC |  |
| qPCR-ULK1 | F: TCGTTGCCTTGTATGACTT | qPCR |
|  | R: TTTATGCGAATGTTGTTGG |  |
| qPCR-KLF4 | F: CGGCAAGACCTACACCAA | qPCR |
|  | R: GATCGGGCAAACTTCCAT |  |
| qPCR-GPI | F: ATTCACTTTGGGAGCAATC | qPCR |
|  | R: ACTCCAACTCTGGCTCAAT |  |
| qPCR-TNNI2 | F: GGGCTCCAAGCACAAGGT | qPCR |
|  | R: GGAAGAGGGTGGTAGTGGC |  |
| qPCR-KLF4-promoter | F: TCAGTTTGTTTTCTCCGTCGCC | qPCR |
|  | R: TTAGCCGCCACCGCCGGTCCTC |  |
| qPCR-GPI-promoter | F: GAGGGAATCTCAGAGGGGAAGG | qPCR |
|  | R: GATCGAGCAGACGTGCAACG |  |
| qPCR-TNNI2-promoter | F: CCCAGCCAAGGGAGGTGCA | qPCR |
|  | R: CTGGCAAGCCGCAAGCAG |  |
| qPCR-CDKN1A-promoter | F: GCGATAAGAGCTGCCGTGC | qPCR |
|  | R: GCGTCACTCTGCGGTTCCC |  |
| qPCR-β-actin | F: GATATTGCTGCGCTCGTTG | qPCR |
|  | R: TTCAGGGTCAGGATACCTCTTT |  |
| 5’ RACE-LncRNA-TBP | Outer: GACCCCAGCTGATCCTGCTTCTGTTCCC | RACE |
| 3’ RACE- LncRNA-TBP | Outer: AGGGGAACAGAAGCAGGATCAGCTGGGG | RACE |
|  | Inner: TGGGTTTCTTCCTCCCTTCACTT |  |
| LncRNA-TBP-ORF1-3xFLAG | F: **AAGCTT**ATGTCCTTTTCCTTCTGCAG | Vector construction |
|  | R: **CTCGAG**GTGCAGAGAAGGAATTATCA |  |
| LncRNA-TBP-ORF2-3xFLAG | F: **AAGCTT** ATGACCGTACCAAAAAAGGA | Vector construction |
|  | R: **AAGCTT**AGACTGTCTGCAAGAAGATA |  |
| LncRNA-TBP-ORF3-3xFLAG | F: **CTCGAG**ATGTGTCATGCAGGTTCCT | Vector construction |
|  | R: **AAGCTT**CTGACACAATTGTAGTTCAAG |  |
| LncRNA-TBP-ORF4-3xFLAG | F: **CTCGAG**ATGCAGGTTCCTGGAAA | Vector construction |
|  | R: **AAGCTT**TGTAGGATACACACATCCG |  |
| LncRNA-TBP-ORF5-3xFLAG | F: **CTCGAG**ATGCAGTTCTCACCGGAT | Vector construction |
|  | R: **AAGCTT**CTCCAGAGTGCTCTCAAATC |  |
| LncRNA-TBP-ORF6-3xFLAG | F: **CTCGAG**ATGTGTGTATCCTACATGAAGG | Vector construction |
|  | R: **AAGCTT**CTGACACAATTGTAGTTCAAG |  |
| LncRNA-TBP-ORF7-3xFLAG | F: **CTCGAG**ATGAAGGGGAACAGAAGCAG | Vector construction |
|  | R: **AAGCTT**CTGACACAATTGTAGTTCAAG |  |
| LncRNA-TBP-ORF8-3xFLAG | F: **CTCGAG**ATGAAGGGGAACAGAAGCAG | Vector construction |
|  | R: **AAGCTT**CTGACACAATTGTAGTTCAAG |  |
| LncRNA-TBP-ORF9-3xFLAG | F: **CTCGAG**ATGAACTTTTCTTACACCCC | Vector construction |
|  | R: **AAGCTT**AGTGAAGGGAGGAAGAAAC |  |
| LncRNA-TBP-ORF10-3xFLAG | F: **CTCGAG**ATGCACAAAGGACTTAATTT | Vector construction |
|  | R: **AAGCTT**AGTGAAGGGAGGAAGAAACC |  |
| LncRNA-TBP-ORF11-3xFLAG | F: **CTCGAG**ATGGGTTTCTTCCTCCCTT | Vector construction |
|  | R: **AAGCTT**CTCAGAGCTCTTGAAAGCAA |  |
| LncRNA-TBP-ORF12-3xFLAG | F: **CTCGAG**ATGTTTCCTGCAGTCAGTAA | Vector construction |
|  | R: **AAGCTT**CTCAGAGCTCTTGAAAGCAA |  |
| pcDNA3.1- LncRNA-TBP | F: **CTCGAG**GAGTGCATGTCCTTTTCCTT | Vector construction |
|  | R: **AAGCTT**GTACCTGTCACTCAGAGCTC |  |
| pcDNA3.1-TBP | F: **CTCGAG**AAGCTTATGGATCAGAACAACAGCT | Vector construction |
|  | R: **AAGCTT**CCTCGAGTTACGTTGTCTTC |  |
| pDC316-RNA2-Puro-LncRNA-TBP | F: **ACTAGT**GAGTGCATGTCCTTTTCCTTCTGCAGTC | Vector construction |
|  | R: **GGATCC**GTACCTGTCACTCAGAGCTCTTGAA |  |

Sequences in bold represent the enzyme cutting sites.

**Supplementary Table 5.** Oligonucleotide sequences in this study.

| **Fragment name** | **Sequences (5’ to 3’)** |
| --- | --- |
| ASO-LncRNA-TBP | CCTCAACATATGCACAAAGG |
| si-TBP | GACTCCAATGACTCCTATA |
